# Functionality of the Na^+^-translocating NADH:quinone oxidoreductase and quinol:fumarate reductase from *Prevotella bryantii* inferred from homology modeling

**DOI:** 10.1007/s00203-023-03769-5

**Published:** 2023-12-21

**Authors:** Jann-Louis Hau, Lena Schleicher, Sebastian Herdan, Jörg Simon, Jana Seifert, Günter Fritz, Julia Steuber

**Affiliations:** 1 Institute of Biology, University of Hohenheim, Garbenstraße 30, 70599 Stuttgart, Germany; 2 HoLMiR-Hohenheim Center for Livestock Microbiome Research, University of Hohenheim, Leonore-Blosser-Reisen-Weg 3, 70599 Stuttgart, Germany; 3 Microbial Energy Conservation and Biotechnology, Department of Biology, Technical University of Darmstadt, Schnittspahnstraße 10, 64287 Darmstadt, Germany; 4 Institute of Animal Science, University of Hohenheim, Emil-Wolff-Straße 8, 70599 Stuttgart, Germany

**Keywords:** Fumarate reduction, Sodium transport, *Prevotella bryantii*, *Segatella bryantii*, *Prevotella bivia*, Menaquinone

## Abstract

Members of the family *Prevotellaceae* are Gram-negative, obligate anaerobic bacteria found in animal and human microbiota. In *Prevotella bryantii*, the Na^+^-translocating NADH:quinone oxidoreductase (NQR) and quinol:fumarate reductase (QFR) interact using menaquinone as electron carrier, catalyzing NADH:fumarate oxidoreduction. *P. bryantii* NQR establishes a sodium-motive force, whereas *P. bryantii* QFR does not contribute to membrane energization. To elucidate the possible mode of function, we present 3D structural models of NQR and QFR from *P. bryantii* to predict cofactor-binding sites, electron transfer routes and interaction with substrates. Molecular docking reveals the proposed mode of menaquinone binding to the quinone site of subunit NqrB of *P. bryantii* NQR. A comparison of the 3D model of *P. bryantii* QFR with experimentally determined structures suggests alternative pathways for transmembrane proton transport in this type of QFR*.* Our findings are relevant for NADH-dependent succinate formation in anaerobic bacteria which operate both NQR and QFR.

**Supplementary Information:**

The online version contains supplementary material available at 10.1007/s00203-023-03769-5.

## Introduction

In the rumen of cattle and sheep, a diverse set of microorganisms provides enzymes to degrade ligno-cellulose material and non-proteinaceous nitrogen (Newbold and Ramos-Morales 2020). In 2015, Henderson and co-workers analyzed 742 ruminal samples from 32 animal species from 35 countries and found a “core microbiota” mainly composed of *Prevotellaceae*, *Butyrivibrio* and *Ruminococcus* species (Henderson et al. 2015). They demonstrated that *Prevotellaceae*, especially *Prevotella ruminicola*, *Prevotella brevis* and *Prevotella bryantii* represent 50–70% of the rumen bacterial population (Gylswyk 1990; White et al. 2014). The genus *Prevotella* has been recently reclassified into seven genera, and *P. bryantii* has been renamed *Segatella bryantii* (Hitch et al. 2022). We are using the traditional nomenclature here, since the reclassification is not translated in the public repositories. With an extensive repertoire of glycoside hydrolases, these bacteria are able to utilize starches, other non-cellulosic polysaccharides and simple sugars as energy sources (White et al. 2014). As an inhabitant of the anoxic ruminal environment, *P. bryantii* conserves energy in the absence of oxygen. Like other *Prevotellaceae*, *P. bryantii* was considered to exclusively perform fermentation (Bryant et al. 1958). During glucose degradation by *P. copri* (Franke and Deppenmeier 2018), 1 mol glucose is converted together with 0.25 mol CO_2_ to 1 mol succinate, 1 mol acetate and 0.24 mol formate. Growth is dependent on CO_2_, indicating that carboxylation of 2-phosphoenolpyruvate (PEP) to oxaloacetate, subsequent formation of fumarate, and reduction of fumarate by quinol:fumarate reductase (QFR) represents the major pathway (Franke and Deppenmeier 2018). This pathway is even more pronounced in CO_2_-dependent degradation of glucose by *P. bryantii* (Schleicher et al. 2021a), with a ratio succinate:acetate = 1:0.5. Likewise, utilization of glucose by the human pathogen *P. bivia* requires CO_2_ and leads to formation of succinate, malate, and acetate in a ratio 1:0.8:0.5. Interestingly, excreted acetate is consumed by *P. bivia* at a later growth stage (Schleicher et al. 2021b).

In a metaproteome study from rumen, Deusch and co-workers identified NQR and QFR from different *Prevotellaceae* and showed that *P. bryantii* NQR acts as sodium-stimulated NADH:quinone oxidoreductase (Deusch et al. 2019). Both NQR and QFR were enriched from membranes and were shown to operate in a respiratory chain, which couples the oxidation of NADH and reduction of fumarate to succinate to the build-up of an electrochemical sodium gradient (Schleicher et al. 2021a). This pointed to an additional mode of energy generation in *P. bryantii* reminiscent to anaerobic respiration, which is catalyzed by NQR and QFR. Likewise, the human pathogen *P. bivia,* which is a causative agent of bacterial vaginosis operates both NQR and QFR and generates a sodium-motive force when grown anoxically on glucose (Schleicher et al. 2021b). In both organisms, menaquinone (MK) was shown to act as electron carrier between NQR and QFR. MK is a low-potential isoprenoid quinone belonging to the subclass of naphthoquinones, characterized by its benzene ring conjugated to the 1,4-benzoquinone structure. MKs exhibit a rather low redox potential (around − 0.07 V) (Magalon and Alberge 2016) and are frequently found in obligate and facultative anaerobic microorganisms. Redox reactions with MK are known for, e.g., hydrogenases, several different NADH dehydrogenases, nitrate reductases, nitrite reductases and fumarate reductases (Simon et al. 2008). Anaerobic respiration utilizing MK was studied in *Wolinella succinogenes*, a rumen bacterium, which relies on QFR, formate dehydrogenase, Ni/Fe hydrogenase, periplasmic nitrate reductase (Nap) or cytochrome *c* nitrite reductase (Nrf) for energy conservation, but does not contain NQR or any other redox Na^+^ pump (Kröger et al. 2002). Accordingly, electron transfer in *W. succinogenes* is coupled to the generation of a proton motive force (PMF) (Kröger et al. 2002). While SQRs/QFRs are present in the three domains of life, NQR is found exclusively in the bacterial kingdom (Reyes-Prieto et al. 2014). NQR evolved from an ancestral ferredoxin:NAD^+^ oxidoreductase (FNO) complex in the Chlorobi/Bacteroidetes group and is only limited distributed across the bacterial lineages (Reyes-Prieto et al. 2014). FNO is a membrane-bound electron transfer complex first identified in *Rhodobacter capsulatus* which was named Rhodobacter Nitrogen Fixation (RNF) complex (Schmehl et al. 1993). FNO/RNF catalyzes the oxidation of ferredoxin and reduction of NAD^+^ generating an electrochemical sodium (or proton) gradient. The complex may also catalyze the reverse reaction, utilizing electrochemical gradients to drive the endergonic reduction of low-potential ferredoxin with NADH. RNF comprises six subunits (RnfABCDEG), of which RnfACDEG are homologous to the NQR subunits NqrEABDC (Buckel 2021; Boiangiu et al. 2005; Biegel et al. 2011; Vitt et al. 2022).

In this study, we explore the mode of function of NQR and QFR from *P. bryantii* using homology modeling and in silico docking. We gain insight into the possible mode of binding of MK to the membrane-bound NqrB subunit of Pb-NQR. A comparative analysis of the modeled Pb-QFR structure with experimentally determined structures of type B QFRs leads to a critical assessment of the E-pathway for transmembrane proton transport. Both NQR and QFR are present in selected organisms from different bacterial phyla, suggesting that sodium-motive NADH:fumarate oxidoreduction is not restricted to the Prevotellaceae family.

## Material and methods

### Protein homology modeling

Structural models of the *P. bryantii* NQR (Pb-NQR) and QFR (Pb-QFR) were generated based on the 3D structures of the highly related NQR from *Vibrio cholerae* (PDB 8A1W, PDB 8ACY) (Hau et al. 2023) and QFR from *Desulfovibrio gigas* (PDB 5XMJ) (Guan et al. 2018)*.* Amino acid sequences of Pb-NQR and Pb-QFR subunits (queries) were retrieved from Uniprot. Structural models of Pb-QFR were generated using Phyre2 (Kelley et al. 2015) and AlphaFold (Jumper et al. 2021), and the resulting models were compared. Subunits of the protein complexes were modeled individually and were superimposed on the template structures. AlphaFold performs structure prediction utilizing neural networks trained by the evolutionary and physical constraints derived from known protein structures. Phyre2 uses an experimentally determined structure of a homolog as a template to predict the structure by performing Hidden-Markov-model (HMM)–HMM matching. This allows for modeling of a protein in the conformation of the respective template. Pb-QFR was modeled in the “normal mode” using a structural template available on the PHYRE2 protein fold recognition server. For modeling of Pb-NQR, “One-to-One threading” was performed using structures of Vc-NQR in the two distinct conformations from the Protein Data Bank (PDB 8A1W, PDB 8ACY). The structural models generated by Phyre2 were compared to models generated by AlphaFold. Note that the Pb-NQR model generated by AlphaFold represents a conformation as observed in the X-ray structure of Vc-NQR (8ACY). The consecutive steps in modeling of protein structures are described in the supplementary material.

### Docking of menaquinone into predicted structure of Pb-NQR

Pb-NQR subunit B was modeled with Phyre2 on the basis of the Vc-NQR structure PDB 8A1W with bound ubiquinone-1. This Pb-NqrB model was used for docking calculations. To avoid blocking of the binding site, rotamers of Glu131 and Tyr133 were aligned to the rotamers of the corresponding amino acids in the *V. cholerae* NQR cryo-EM structure (PDB 8A1W) using COOT 0.9.8.8. Docking was performed using OEDOCKING 4.1.1.0. OpenEye, Cadence Molecular Sciences, Inc., Santa Fe, NM (http://www.eyesopen.com). Pb-NQR structure was prepared for docking by applying a 13 × 23 × 16 Å box over the quinone binding site and creating a contour of the binding site (4940 Å^3^) using the Make Receptor GUI with balanced settings. Menaquinone-1 was prepared for docking from SMILES using OMEGA 4.1.2.0 (Hawkins et al. 2010). Menaquinone-1 was docked into Pb-NQR with FRED (McGann 2011, 2012).

### Identification of NQR, RNF and QFR

Representative organisms from selected bacterial phyla were analyzed, which contained succinate:quinone reductases and/or quinol:fumarate reductases as demonstrated by experimental data. We also searched for NQR and RNF by aligning the amino acid sequences of the NQR subunits from *Vibrio cholerae* serotype O1 (strain ATCC 39541) and of RnfB from *Escherichia coli* K12 to the corresponding genomes using the basic local alignment search tool BLAST (Mount 2007). Sequences of subunits of enzymes were retrieved from Uniprot (https://www.uniprot.org/).

## Results and discussion

To gain knowledge on the possible functions of *P. bryantii* NQR and QFR, structural models were obtained by comparisons with the NQR from *Vibrio cholerae* (Steuber et al. 2014; Hau et al. 2023), and QFRs from *W. succinogenes* (Madej et al. 2006) and *Desulfovibrio gigas* (Guan et al. 2018).

### 3D structural model of QFR from *P. bryantii*

**Fig. 1:**
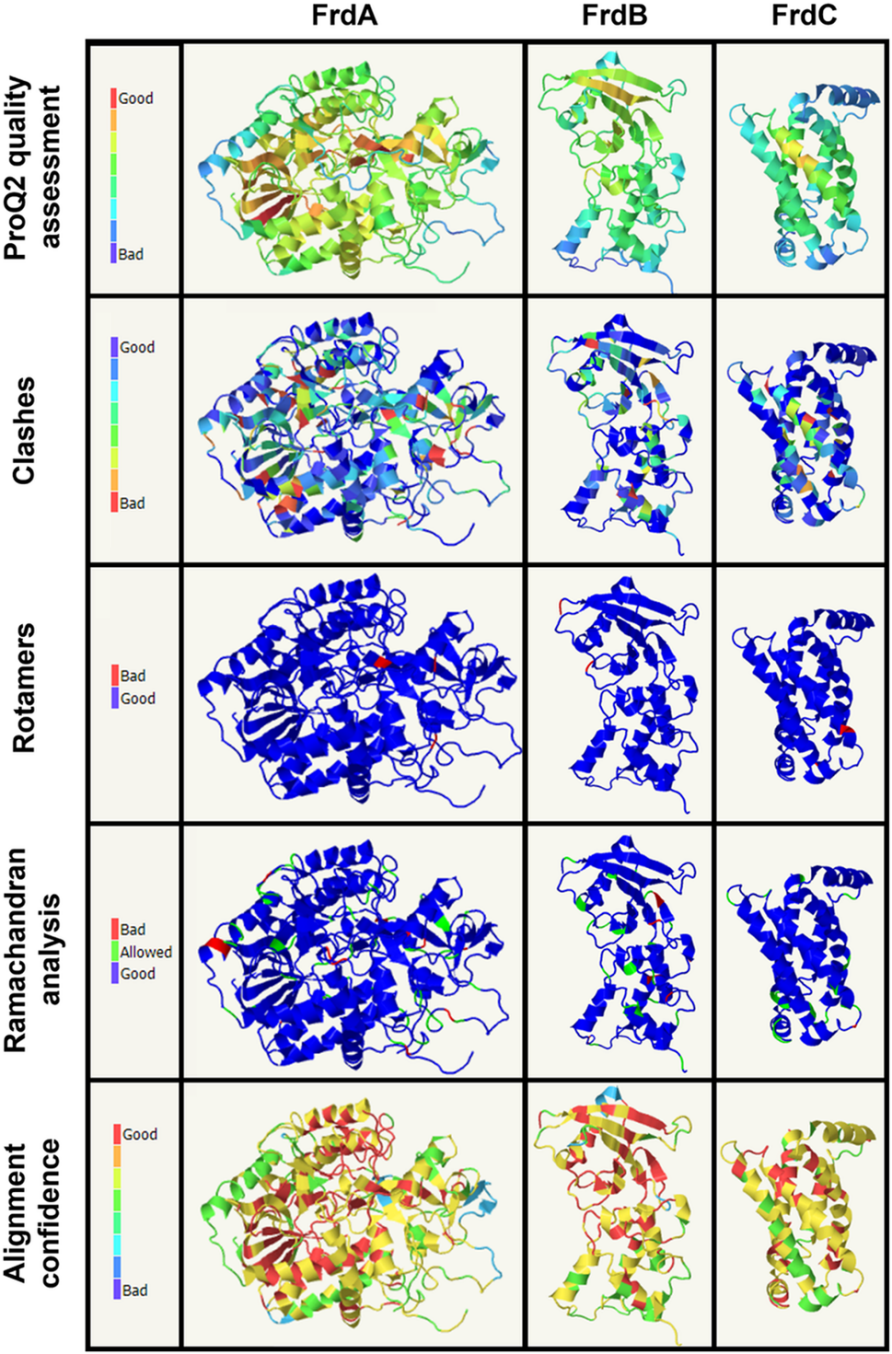
Homology modeling of the FrdA (second column), FrdB (third column) and FrdC (fourth column) subunits from *P. bryantii* QFR performed by Phyre2, using the *D. gigas* QFR subunits as templates. Shown are the ProQ2 quality assessment (first row), clash analyses (second row), rotamer analyses (third row), Ramachandran analyses (fourth row) and alignment confidence (fifth row). The results of the different analyses are color-coded (first column)

**Fig. 2:**
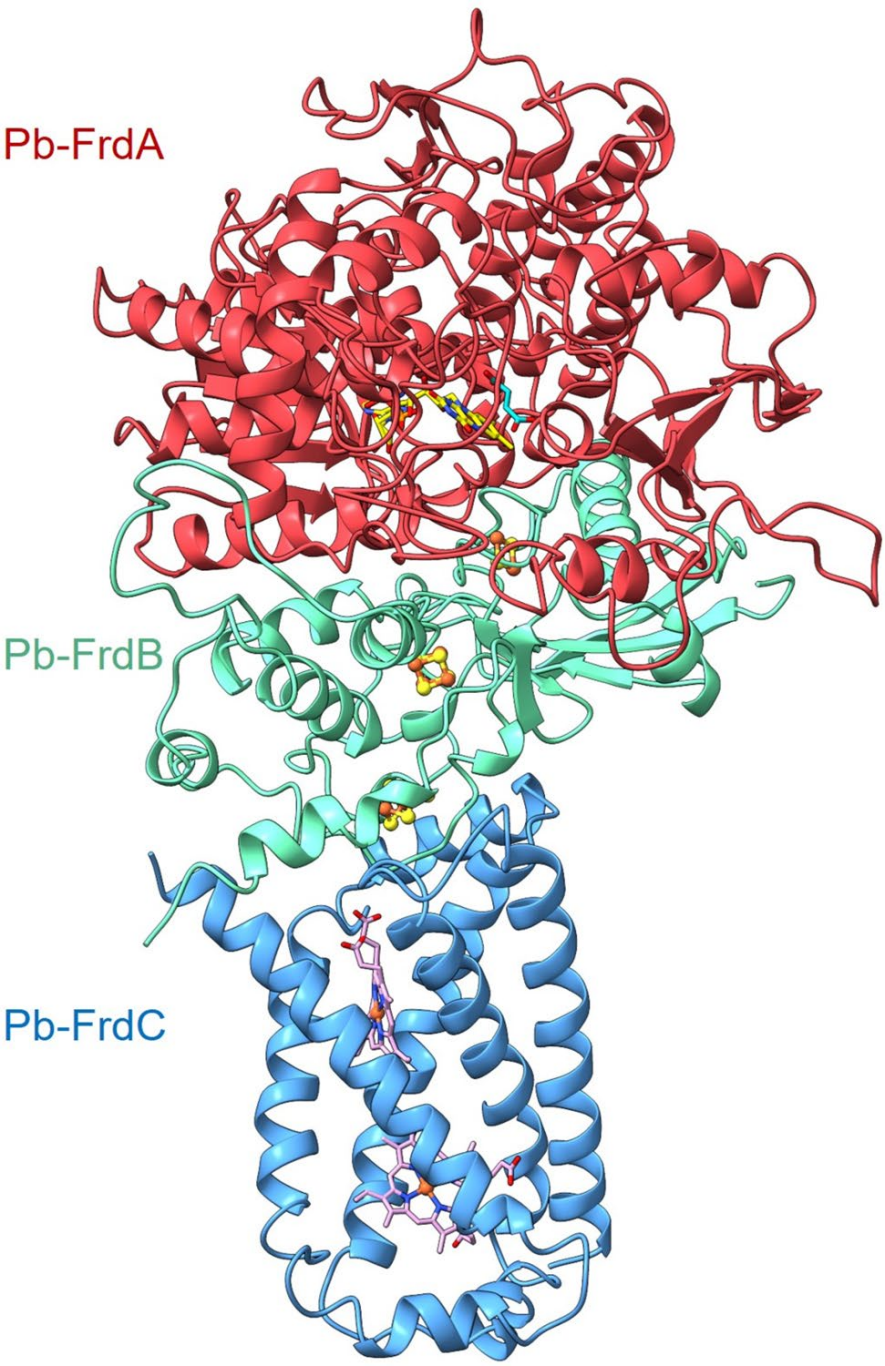
Modeled structure of *P. bryantii* QFR. The QFR is composed of 3 subunits: FrdA (red), FrdB (green) and FrdC (blue). Cofactors were positioned according to their localization in the *D. gigas* QFR. These are one FAD in FrdA, three FeS clusters in FrdB and two hemes *b* in FrdC. Flavins are indicated as yellow sticks, iron atoms are shown as orange spheres and sulfur atoms as yellow spheres, and hemes *b* are represented as pink sticks. The Pb-QFR structure was modeled with Phyre2, using the *D. gigas* crystallographic structure as template. Picture was created with UCSF ChimeraX version 1.5

The model of the *P. bryantii* QFR (Pb-QFR) was built based on the structure of the *Desulfovibrio gigas* QFR (Dg-QFR) (Guan et al. 2018). This model was aligned with the QFR of *W. succinogenes* (Ws-QFR). The Ws-QFR is a type B quinol:fumarate reductase, oxidizing MKH_2_ and reducing fumarate to succinate. It is composed of three subunits (FrdABC), with FrdA (~ 75 kDa) containing one FAD, FrdB (~ 27 kDa) harboring three FeS centers and FrdC (~ 30 kDa) comprising two hemes *b* (Lancaster et al. 1999). Ws-QFR forms a homodimer in crystals with a dimension of 75 Å × 120 Å × 50 Å (Lancaster et al. 1999). The catalytic site for MKH_2_ oxidation, and therefore the entry site of the electrons into the protein, is located in FrdC. After oxidation of MKH_2_ by the hemes *b* in FrdC, the electrons are transferred via the proximal heme *b* and the FeS clusters of FrdB to the covalently bound FAD, and finally, fumarate is reduced to succinate (Lancaster et al. 1999). The 3D crystallographic structure of the Dg-QFR served as template for model building of Pb-QFR by Phyre2 (Kelley et al. 2015). Here, a high confidence, coverage and TM score were calculated, despite moderate overall sequence identities (supplementary material table S3). Next, a more detailed analysis of the secondary structure quality of the modeled QFR structure was performed. The ProQ2 assessment tool implemented in Phyre2 indicates a good quality of the core structures, illustrated by yellow and orange colored cores of the modeled protein structures (Fig. 1, first row). Blue color indicates regions where the quality of the model is lower, which is frequently observed in loops or other connecting protein domains. The clash analysis indicates that only a few domains in the models exhibit unfavorable positions of amino acid side chains. Thus, an overall correct positioning of side chains in the models is assumed (Fig. 1, second row). Furthermore, only a few domains reveal incorrect modeling in terms of rotamer analysis (Fig. 1, third row). Ramachandran analysis reveals only a few disallowed regions near or in the loop domains in the modeled FrdABC structures (Fig. 1, fourth row). The models obtained by Phyre2 and AlphaFold were virtually identical. In summary, the overall stereochemistry and geometry of the structural models of Pb-QFR subunits are adequate, with the exception of few loop regions, which exhibit poor alignment confidence (Fig. 1, fifth row). The model of the Pb-QFR complex was built based on the models of the three Frd subunits (Fig. 2). This model includes FrdA (red), FrdB (green) and FrdC (blue), and six cofactors known from the *D. gigas* enzyme, which were placed at corresponding positions into the Pb-QFR structural model. Hypothetically, FrdA contains one covalently bound FAD, FrdB comprises three FeS clusters and FrdC harbors two hemes *b*. Since the *W. succinogenes* QFR was characterized in terms of both structure and function (Lancaster et al. 2000; Lancaster 2002), the Pb-QFR structural model was compared with the crystallographic structure of the Ws-QFR (PDB 2BS2) to search for conserved amino acid residues, which are likely to be important for substrate conversion, electron and proton transfer.

### Predicted mode of operation of QFR from *P. bryantii* based on the structural model

**Fig. 3:**
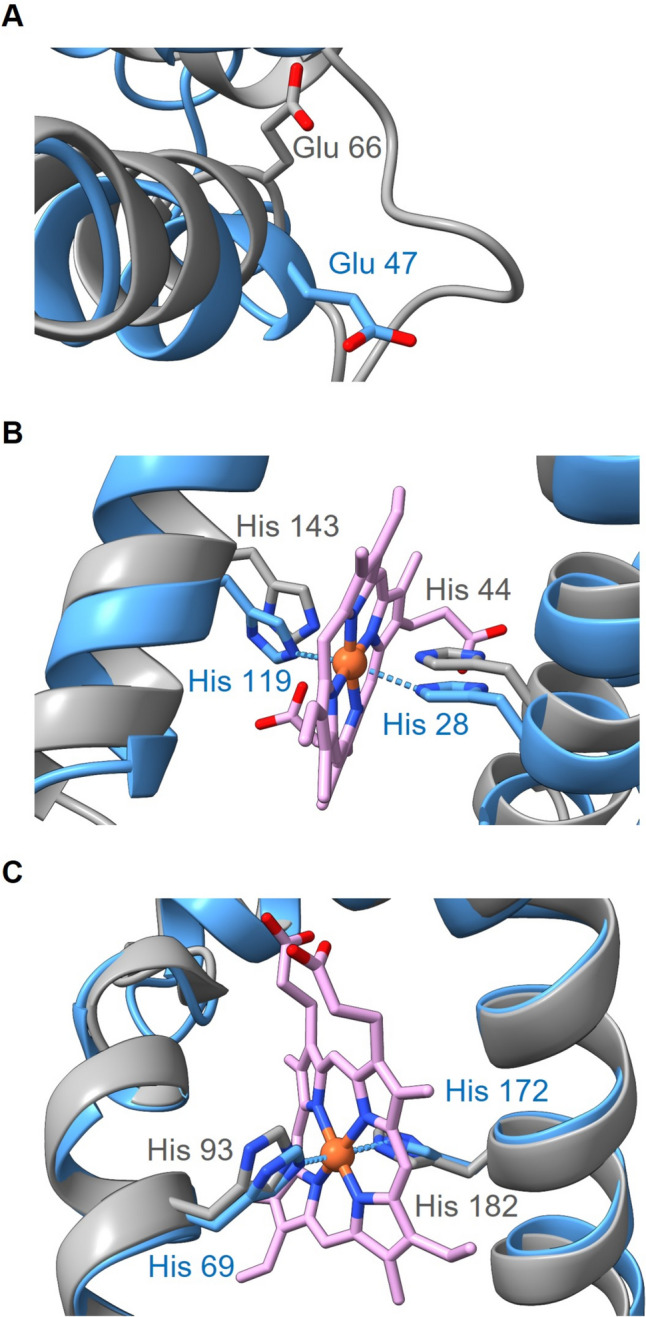
Structural alignment of the model of FrdC from *P. bryantii* (blue) and the structure of FrdC from *W. succinogenes* (gray). **A** Close-up view of the menaquinol oxidation site, with Glu66 from Ws-FrdC and Glu47 from Pb-FrdC, which may be part of a proton wire toward the periplasm. **B** Close-up view of distal heme *b*, coordinated by His44 and His143 in Ws-FrdC, and by His28 and His119 in Pb-FrdC. **C** Coordination of proximal heme *b* in a close-up view. His93 and His182 are responsible for heme *b* coordination in Ws-FrdC. His 69 and His172 function as heme *b* ligands in Pb-FrdC. Heme *b* structures are indicated in pink. The coordinates of the Ws-FrdC structure were obtained from the protein data bank (PDB 2BS2) and the Pb-FrdC, based on the *D. gigas* crystallographic structure, was modeled with Phyre2. The structures were aligned and drawn with UCSF ChimeraX version 1.5

The binding site for MK in Ws-QFR is suggested to be near the distal heme in FrdC, with Glu66 playing an essential role for MKH_2_ oxidation (Lancaster et al. 2000) (Fig. 3A). In the modeled Pb-FrdC, a glutamate is predicted in this region (Glu47) but at a position shifted by three amino acid residues (Fig. 3A). Still, the corresponding Glu47 from Pb-FrdC is predicted to be located in a cavity leading to the periplasm, as observed with Glu66 in Ws-FrdC. Glu47 is predicted in a putative loop region of Pb-FrdC, which exhibits only low sequence similarity to the corresponding region in the FrdC from *D. gigas*, according to the Phyre2 qualification analysis of the modeled Pb-FrdC structure (Fig. 1). In *W. succinogenes* FrdC, Glu66 accepts protons from quinol and releases them to the periplasm (Lancaster et al. 2000), and a similar function seems likely for Pb-Glu47. Electrons from MKH_2_ are transferred to the distal heme *b*, which is coordinated by His44 and His143 in Ws-FrdC (Fig. 3B) (Lancaster et al. 1999). A second, proximal heme *b* in Ws-FrdC enables electron transfer from the FrdC to the FrdB subunit, facilitated by its location toward the cytoplasmic surface of the membrane and thus to the iron-sulfur clusters of Ws-FrdB. This proximal heme *b* is also coordinated by two histidines (His93, His182) (Fig. 3C) (Lancaster et al. 1999). The modeled Pb-FrdC structure comprises five transmembrane helices, containing these four histidines in the modeled core structure at the very same positions with the same orientations (His28 and His119 for distal heme *b*; His69 and His172 for proximal heme *b*) (Fig. 3B and C).

**Fig. 4:**
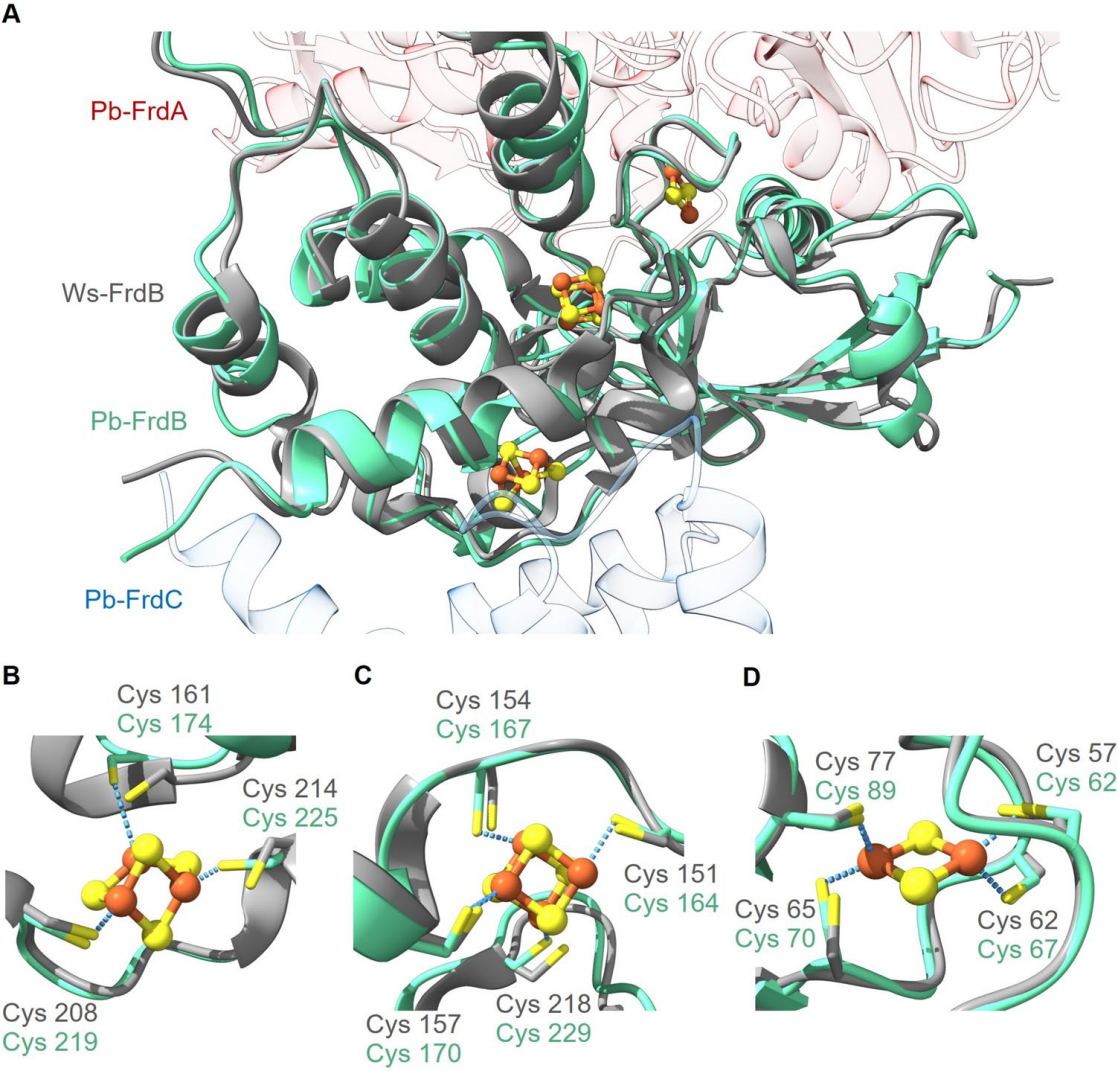
Structural alignment of the model of FrdB from *P. bryantii* (green) and the structure of FrdB from *W. succinogenes* (gray). **A** FrdB is located between FrdA and FrdC, and harbors three FeS clusters ([3Fe–4S], [4Fe–4S], [2Fe–2S]). **B** Close-up view of the [3Fe–4S] cluster. In Ws-FrdC (PDB 2BS2), it is coordinated by Cys161, Cys208 and Cys214. In Pb-FrdC, it is predicted to be ligated by Cys174, Cys219, Cys225. **C** Close-up view of the [4Fe–4S] cluster. In Ws-FrdC, the residues of Cys151, Cys154, Cys157 and Cys218 are responsible for FeS ligation. In Pb-FrdC Cys164, Cys167, Cys170 and Cys229 are predicted to coordinate the FeS cluster. **D** Close-up view of the [2Fe–2S] cluster with Cys57, Cys62, Cys65 and Cys77 acting as ligands in Ws-FrdC. Cys62, Cys67, Cys70 and Cys89 are predicted to coordinate the [2Fe–2S] cluster in Pb-FrdB. Cys70 was adjusted to the rotamer observed in Cys65 in Ws-FrdB. Fe atoms are indicated as orange spheres, sulfur atoms as yellow spheres and thiol groups of cysteines as yellow sticks. The coordinates of the Ws-FrdB structure were obtained from the protein data bank (PDB 2BS2) and the Pb-FrdB, based on the *D. gigas* crystallographic structure, was modeled with Phyre2. The structures were aligned and drawn with UCFS ChimeraX version 1.5

The [3Fe–4S] cluster, proximal to heme *b,* is coordinated by the thiol groups of the three cysteines Cys161, Cys208 and Cys214 (Fig. 4B). These amino acid residues are located in a domain, which is in contact with the FrdC subunit. The [3Fe–4S] cluster accepts electrons from the proximal heme *b* and passes them to the [4Fe–4S], which is ligated by four cysteine residues (Cys151, Cys154, Cys157, Cys218) (Fig. 4C). Electron transfer proceeds via [2Fe–2S] cluster, located between the [4Fe–4S] cluster and the Ws-FrdA subunit. The [2Fe–2S] cluster is coordinated by four cysteine residues (Cys57, Cys62, Cys65, Cys77) (Fig. 4D). In the predicted model of Pb-FrdB, all cysteine residues required for the coordination of the three FeS centers are present and oriented in a manner, which would permit Fe ligation by the thiol groups (Fig. 4). Thus, it can be predicted that Cys174, Cys219 and Cys225 are responsible for [3Fe–4S] coordination (Fig. 4B), and that Cys164, Cys167, Cys170 and Cys229 ligate the [4Fe–4S] (Fig. 4C). The [2Fe–2S] cluster is predicted to be coordinated by Cys62, Cys67, Cys70 and Cys89 (Fig. 4D).

**Fig. 5:**
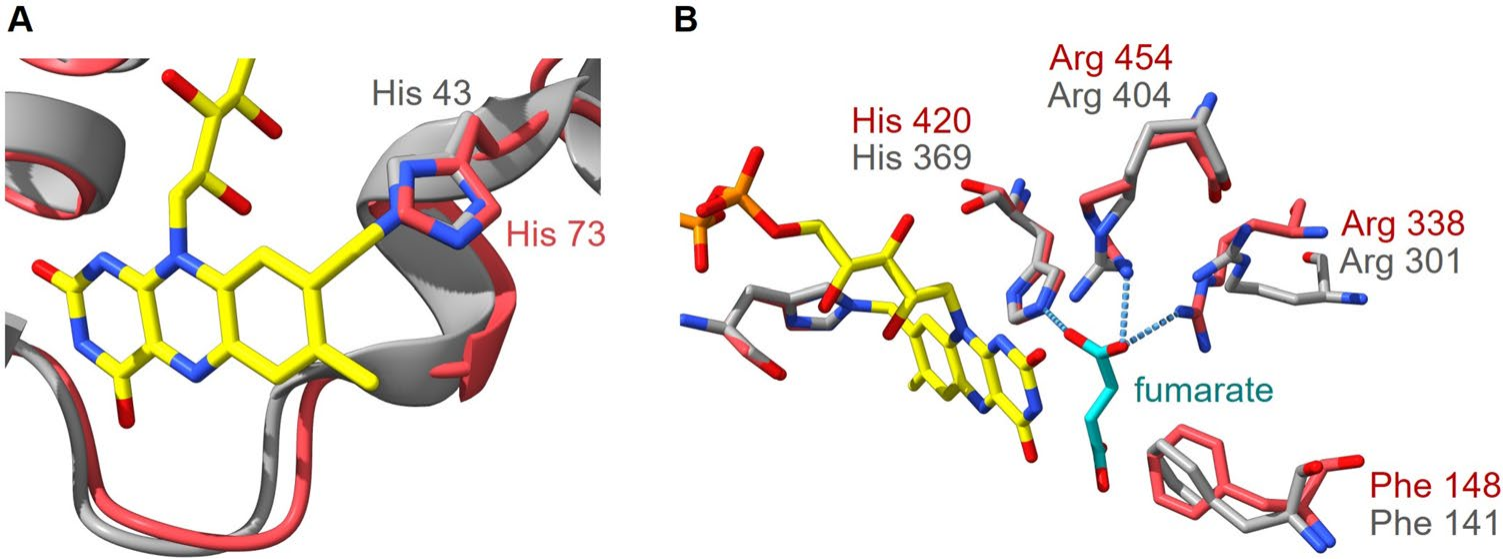
Structural alignment of the model of FrdA from *P. bryantii* (red) and the structure of FrdA from *W. succinogenes* (gray). **A** Close-up view of FAD-binding site with His43 responsible for covalent attachment of FAD in *W. succinogenes* FrdA. The corresponding His73 in the model of Pb-FrdA adopts a very similar position. **B** Close-up view of the dicarboxylate-binding site, formed by Phe141, Arg301, His369 and Arg404 in Ws-FrdA, and by Phe148, Arg338, His420 and Arg454 in the model of Pb-FrdA. FAD is indicated in yellow and fumarate as cyan sticks. The coordinates of the Ws-FrdA structure were obtained from the protein data bank (PDB 2BS2) and the Pb-FrdA structure, based on the *D. gigas* crystallographic structure, was modeled with Phyre2. Picture was created with UCFS ChimeraX version 1.5

From Ws-FrdB, electrons are transferred to a covalently bound FAD in Ws-FrdA. This FAD is linked by its 8α-methyl group to the histidine His43 (Fig. 5A) (Lancaster et al. 1999). The modeled Pb-FrdB structure predicts with a high confidence also a histidine (His73) at the same position with the same orientation. The Ws-FrdA subunit also comprises a dicarboxylate-binding site for binding of fumarate. This binding site is formed mainly by the isoalloxazine ring of the FAD and four crucial amino acid residues (Phe141, Arg301, His369, Arg404) (Fig. 5B) (Lancaster et al. 1999). Analyzing the modeled Pb-FrdA structure, these four residues are also predicted (Phe148, Arg338, His420, Arg454) in a region with a high prediction quality (Fig. 5B).

The model of Pb-QFR is in accordance with experimental data (Schleicher et al. 2021a) confirming the existence of a *P. bryantii* QFR composed of three subunits (FrdABC), with similar catalytic functions as described for *W. succinogenes* (Lancaster et al. 2000, 2005; Lancaster 2013). Although some domains of the Pb-QFR model are predicted at moderate confidence (Fig. 1), the core regions of the model structure responsible for binding of cofactors and substrates are predicted with high confidence and display with a high degree of conservation among QFRs from *D. gigas* and *W. succinogenes.* This supports the assumption that *P. bryantii* operates a QFR belonging to the type B of SQRs. The Pb-QFR model substantiates the biochemical studies (Schleicher et al. 2021a) and provides a good starting point for further mechanistic studies on Pb-QFR.

**Fig. 6:**
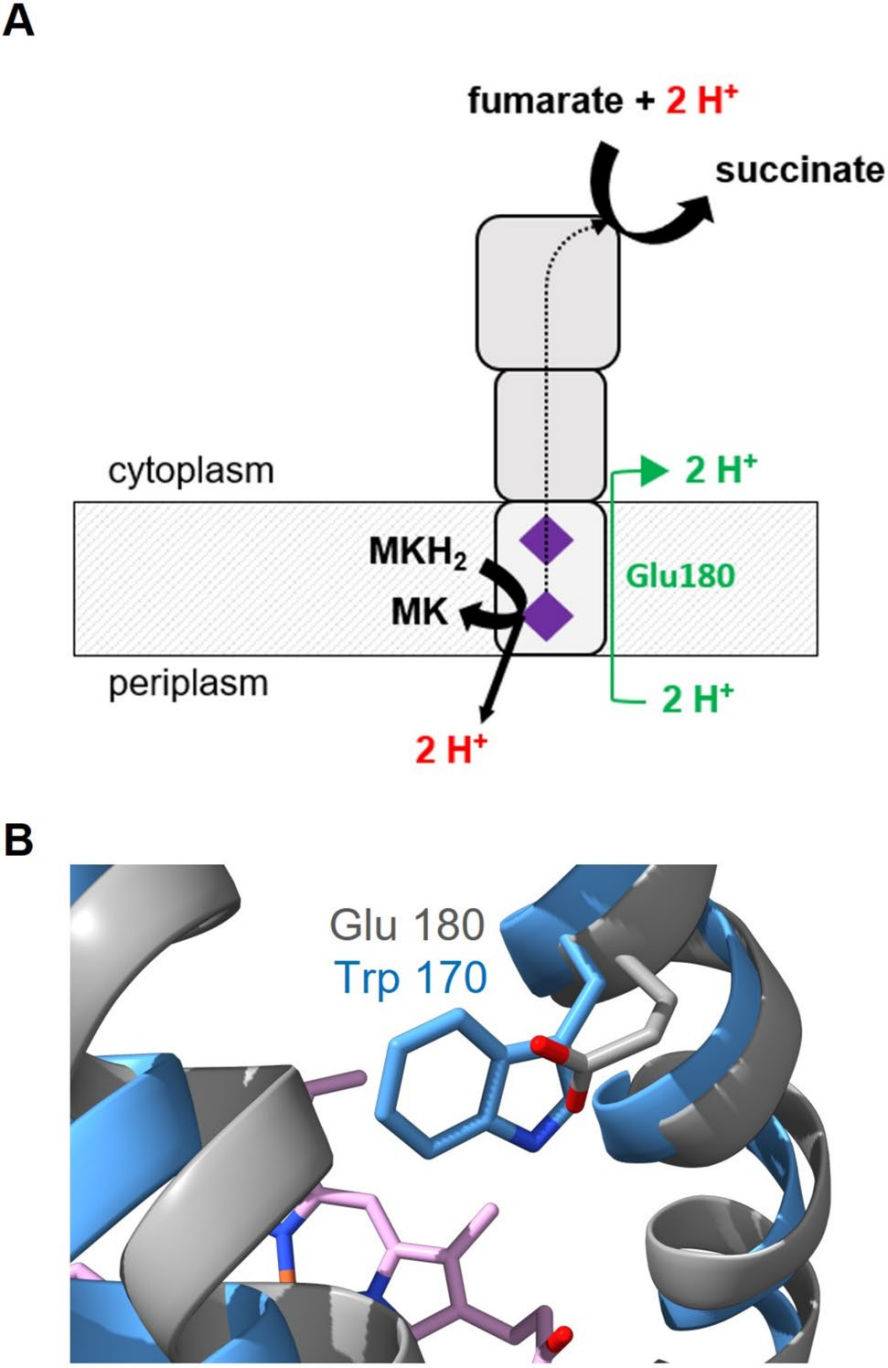
Operational mode of the quinol:fumarate oxidoreductase from *W. succinogenes* and comparison with the *P. bryantii* enzyme. **A** Electroneutral quinol:fumarate oxidoreduction by Ws-QFR requires proton transport from the periplasm into the cytoplasm via the E-pathway involving Glu180 (indicated in green). The inner bacterial membrane is indicated with gray stripes. Upper part represents the cytoplasm, lower part represents the periplasm. Hemes in FrdC are indicated as purple diamonds. *MK* menaquinone, *MKH*_*2*_ menaquinol. Figure was adapted from Madej et al. (2006). **B** Comparison of the FrdC region comprising the Glu180 in *W. succinogenes* (gray) and Trp170 in the model of FrdC from *P. bryantii* (blue). Heme *b* structure is indicated in pink. The coordinates of the Ws-FrdC structure were obtained from the protein data bank (PDB 2BS2). Pb-FrdC was modeled with Phyre2 based on the *D. gigas* crystallographic structure. Picture was created with UCFS ChimeraX version 1.5

In fumarate respiration, two protons are liberated by oxidation of MKH_2_ and two protons are consumed by reduction of fumarate to succinate. The overall reaction contributes to the build-up of an electrochemical proton gradient (H^+^/e^−^ = 1), if protons from MKH_2_ are released at the periplasmic aspect of the membrane. In contrast, if protons liberated during oxidation of MKH_2_ are released at the cytoplasmic side, QFR would operate in an electroneutral manner. For QFR of *W. succinogenes*, it was shown that protons are released into the periplasm, with an essential role for Glu66 acting as proton acceptor during MKH_2_ oxidation. Glu66 is located in a cavity connected to the periplasmic aqueous phase (Lancaster et al. 2005). Nevertheless, it was demonstrated that in *W. succinogenes*, the QFR reaction is electroneutral (Kröger et al. 2002). In a counterbalanced mechanism of H^+^ movement, two protons are transferred from the periplasm into the cytoplasm via the so-called E-pathway in concert with the two electrons passing from MKH_2_ via the two heme groups to fumarate (Fig. 6A) (Kröger et al. 2002). For *W. succinogenes* QFR, it was shown that Glu180 plays a crucial role in this transmembrane proton transport through the E-pathway (Madej et al. 2006). In Pb-QFR, protons from MKH_2_ oxidation are likely to be released into the periplasm, considering the 3D structural model of Pb-QFR. However, the predicted structure of Pb-FrdC does not confirm a similar position of a Glu at the corresponding position. Thus, an obvious H^+^ acceptor/donor as identified in the E-pathway of Ws-FrdC is absent in Pb-FrdC (Fig. 6B). Notably, highly similar FrdC subunits from at least four *Prevotellaceae* species also lack a protonatable amino acid residue at the corresponding position (supplementary material fig. S1), indicating that the E-pathway proposed for *W. succinogenes* QFR is not strictly retained in QFRs from *Prevotellaceae*. A tyrosine (Tyr42) near the distal heme in Pb-FrdC is reminiscent to Tyr63 observed in the 3D structure of *Desulfovibrio gigas* FrdC (Guan et al. 2018). QFR of *D. gigas* is predicted to facilitate compensatory proton transport, with Tyr63 proposed to act as initial proton acceptor (Guan et al. 2018). In Ws-FrdC, the carboxylic group of the propionate side chain of ring C from the distal heme is considered to act as proton donor/acceptor during proton transport via the E-pathway (Lancaster et al. 2000). In the modeled Pb-FrdC structure, the propionate side chain of the distal heme assumes a very similar position (Fig. 3). NADH:fumarate oxidoreduction in *P. bryantii* results in the build-up of a sodium-motive, but not of a proton-motive force (Schleicher et al. 2021a). The 3D structural model supports the notion that fumarate reduction by Pb-QFR does not result in the build-up of a proton gradient due to counter-transport of protons, but the identification of the proton pathway requires further investigations.

### 3D structural model of Pb-NQR

*P. bryantii* critically depends on primary Na^+^ transport to energize the membrane, which is catalyzed by a sodium-translocating NADH:quinone oxidoreductase (NQR) (Schleicher et al. 2021a). The structure of Na^+^-translocating NQR from *Vibrio cholerae* has been studied intensively and was used to build a reliable structural model of Pb-NQR. Vc-NQR comprises six subunits (NqrABCDEF) and harbors six cofactors: one FAD in NqrF, two [2Fe–2S] clusters in NqrF and between NqrD/E, one riboflavin in NqrB, and two covalently bound FMNs in NqrB and NqrC (Hau et al. 2023; Kishikawa et al. 2022). The subunit NqrF represents the entry site of the electrons from NADH into the complex, thus catalyzing the NADH dehydrogenase reaction. NqrF is composed of two hydrophilic domains, the N-terminal [2Fe–2S] ferredoxin-like domain and the C-terminal FAD-binding domain, and it is anchored into the NQR complex by one single transmembrane helix. NADH is oxidized by FAD and the electrons are transferred to the [2Fe–2S] in the ferredoxin-like domain (Hau et al. 2023; Steuber et al. 2014; Türk et al. 2004). FAD transiently forms a neutral flavosemiquinone during the electron transfer to [2Fe–2S] that serves as a one-electron acceptor. Subsequently, the electrons are transferred from the NqrF ferredoxin-like domain to an electron transfer chain comprising an intramembranous [2Fe–2S] cluster located in the membrane subunits NqrD and NqrE, an FMN in periplasmic subunit NqrC, and FMN and riboflavin in subunit NqrB. In a last step, electrons are transferred from riboflavin to substrate UQ-8 in NqrB, resulting in formation of UQH_2_. In Pb-NQR, MK acts as ultimate electron acceptor (Schleicher et al. 2021a).

The membrane-bound Vc-NQR complex has dimensions of about 90 Å × 140 Å × 52 Å and an overall molecular mass of 220 kDa (NqrA: 48.6 kDa, NqrB: 45.3 kDa, NqrC: 27.6 kDa, NqrD: 22.8 kDa, NqrE: 21.4 kDa and NqrF: 45.1 kDa). Vc-NQR adopts distinct conformational states, which are crucial for the redox-driven Na^+^ translocation (Hau et al. 2023). To gain insight into the mode of operation of Pb-NQR, we created models of two states of the catalytic cycle as observed in Vc-NQR (PDB 8ACY and 8A1W) (Hau et al. 2023). Creating such specific models requires a template-based homology modeling approach like in Phyre2, since AlphaFold creates a single model based on all available structures. The models of Pb-NQR created with Phyre2 were highly similar to the corresponding structures of Vc-NQR displaying RMSDs for the Cα atoms of 0.02–0.305 Å for one template (PDB 8ACY) and 0.01–0.26 Å for the second template (PDB 8A1W), respectively. AlphaFold created a model of Pb-NQR that reflects only one conformation. Overall, one conformational model created with Phyre2 and the model created by AlphaFold were highly similar. Subtle differences between both models were observed in subunit Pb-NqrE. Pb-NqrE exhibits an extension of 10 residues compared to Vc-NqE, which are modeled by AlphaFold as an extended periplasmic loop (residues 133–146), while the model of Pb-NqrE places this region into the transmembrane region, which could result in sterical clashes with subunit NqrD. Evaluation of both models and pairwise-alignment favor the model created by AlphaFold. Further minor negligible differences between both models were observed for some solvent-exposed loops, which are not relevant for binding of cofactors or substrates and are not involved in conformational changes.

**Fig. 7:**
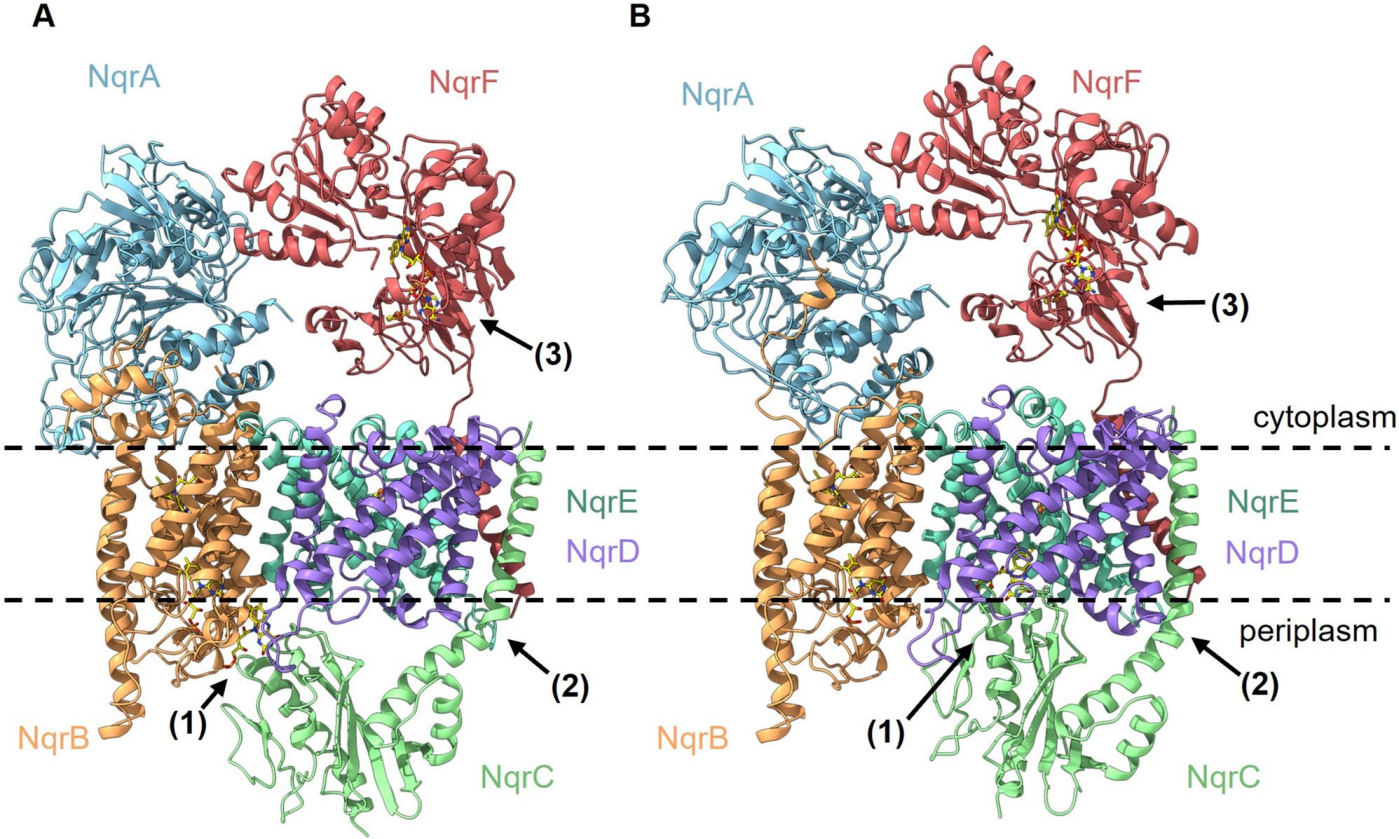
Modeled structures of NQR from *P. bryantii* in two conformations. The NQR is composed of six subunits: NqrA (blue), NqrB (orange), NqrC (green), NqrD (purple), NqrE (cyan) and NqrF (red). **A** Structural model of Pb-NQR based on Vc-NQR (PDB 8A1W). **B** Structural model of Pb-NQR based on Vc-NQR (PDB 8ACY). The models include the cofactors (one FAD and one [2Fe–2S] in NqrF, one [2Fe–2S] ligated by NqrD and NqrE, one FMN in NqrC, one FMN and one riboflavin in NqrB). Flavins are indicated in yellow, iron atoms are shown as orange spheres, sulfur atoms as yellow spheres, and the membrane is indicated with dashed lines. A comparison of the two Pb-NQR structural models (**A** and **B**) reveals domains which exhibit different conformations, in accord with the corresponding structures of Vc-NQR used for model building. Predicted changes in conformation (highlighted by black arrows) affect (1) the position of the periplasmic FMN domain of NqrC which is either oriented toward NqrB (**A**) or NqrD/E (**B**); (2) the position of the single transmembrane helix of NqrC in the two conformations (**A**) and (**B**), respectively; (3) the position of the ferredoxin-like domain of NqrF with its [2Fe–2S] cluster in proximity to the membrane (**A**) or remote from the membrane (**B**). Pb-NQR structures were modeled with Phyre2 and the structures were drawn with UCFS ChimeraX version 1.5

Figure 7 depicts the two conformations modeled for Pb-NQR. Each model of Pb-NQR comprises all six subunits NqrA-F and all cofactors: NqrF comprises one FAD and one [2Fe–2S] cluster, NqrD/E contain one [2Fe–2S], NqrC harbors one FMN, and NqrB comprises one FMN and one riboflavin. The structures of Vc-NQR (Hau et al. 2023; Steuber et al. 2014) reveal that NQR undergoes drastic structural changes during the catalytic cycle involving at least subunits NqrF, NqrC, NqrD and NqrE. The two distinct structural models of Pb-NQR reflect two different states of the Pb-NQR and support that Pb-NQR also functions as a conformationally driven redox pump (Fig. 7).

### Redox cofactor-binding sites in Pb-NQR

**Fig. 8:**
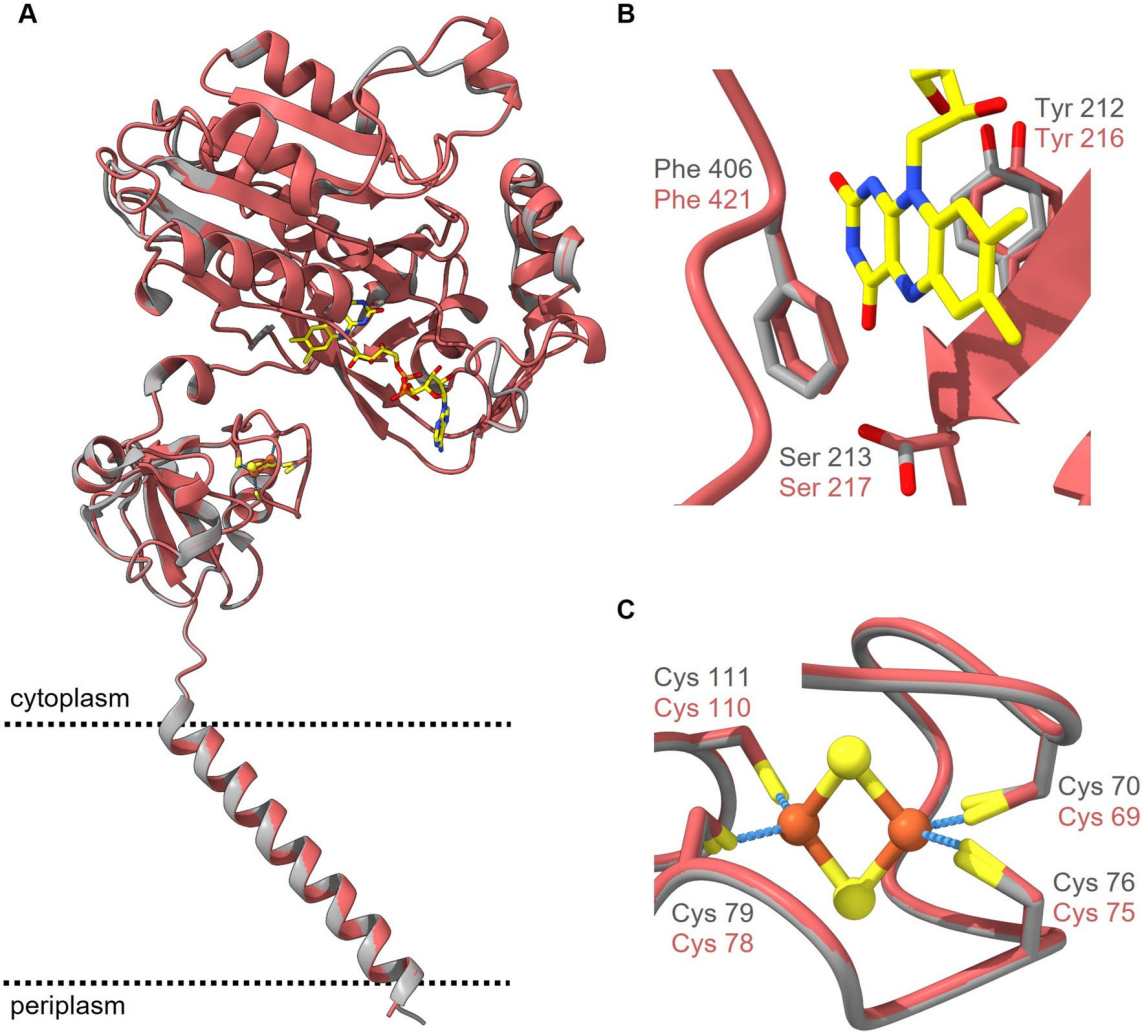
Structural alignment of the model of subunit NqrF from *P. bryantii* (red) and the structure of subunit NqrF from *V. cholerae* (gray, PDB 8A1W). **A** Subunit NqrF with two cytoplasmic domains harboring the FAD and [2Fe–2S] cofactors, respectively. **B** Close-up view of the FAD-binding domain. The conserved amino acids Tyr212, Ser213 and Phe406 in Vc-NqrF and Tyr216, Ser217 and Phe421 in Pb-NqrF coordinate the non-covalently bound FAD. **C** Close-up view of the [2Fe–2S] coordination. Four cysteines (Cys70, Cys76, Cys79 and Cys111 in Vc-NqrF; Cys69, Cys75, Cys78 and Cys110 in Pb-NqrF) coordinate the [2Fe–2S] with their sulfur residues (yellow-colored sticks). Cysteine rotamers of Pb-NqrF were adjusted to match the rotamers observed in Vc-NqrF. FAD is indicated in yellow, iron atoms are shown as orange spheres, and sulfur atoms as yellow spheres. The coordinates of the Vc-NqrF structure were obtained from the protein data bank (PDB 8A1W) and the Pb-NqrF structure was modeled with Phyre2. The structures were aligned and drawn with UCFS ChimeraX version 1.5

The cofactors in Vc-NQR define an electron transfer pathway that passes two times the cytoplasmic membrane (Hau et al. 2023; Steuber et al. 2014). Thus, information on the presence and position of the cofactors are important to evaluate whether such an electron transfer pathway occurs also in Pb-NQR. However, most modeling software suites do not place automatically the corresponding cofactors found in related proteins. Therefore, the cofactors in Pb-NQR were placed by manual model building after critical assessment of the residues in the cofactor-binding sites. The redox cofactor NADH is oxidized in subunit NqrF by the cofactor FAD (Steuber et al. 1997; Türk et al. 2004). The high-resolution structure of Vc-NqrF revealed the binding of NADH (Hau et al. 2023) and a critical role of the C-terminal residue Phe406 in NAD^+^ release. Pb-NqrF exhibits reasonable sequence similarity to Vc-NqrF; nevertheless, important features are highly conserved in Pb-NqrF and the 3D models show a conserved binding site for cofactor FAD and suggest a highly similar binding mode for NADH (Fig. 8A). In particular, all residues in the active site of the Pb-NqrF model display very similar positions compared to Vc-NqrF. In Vc-NqrF, three residues are in close proximity of the isoalloxazine moiety of FAD: Tyr212, Ser213 and Phe406 (Hau et al. 2023) (Fig. 8B). In the models of Pb-NqrF, the corresponding amino acid residues Tyr216, Ser217, Phe421 are at identical positions and support that FAD and NADH bind as observed in Vc-NqrF (Fig. 8B). In the N-terminal ferredoxin-like domain of Vc-NqrF, the [2Fe–2S] cluster is coordinated by the four cysteine residues Cys70, Cys76, Cys79 and Cys111 (Hau et al. 2023) (Fig. 8C), which are strictly conserved in Pb-NqrF. The 3D model showed that the corresponding residues Cys69, Cys75, Cys78, Cys110 reside in very similar positions and can accommodate an [2Fe–2S] center.

**Fig. 9:**
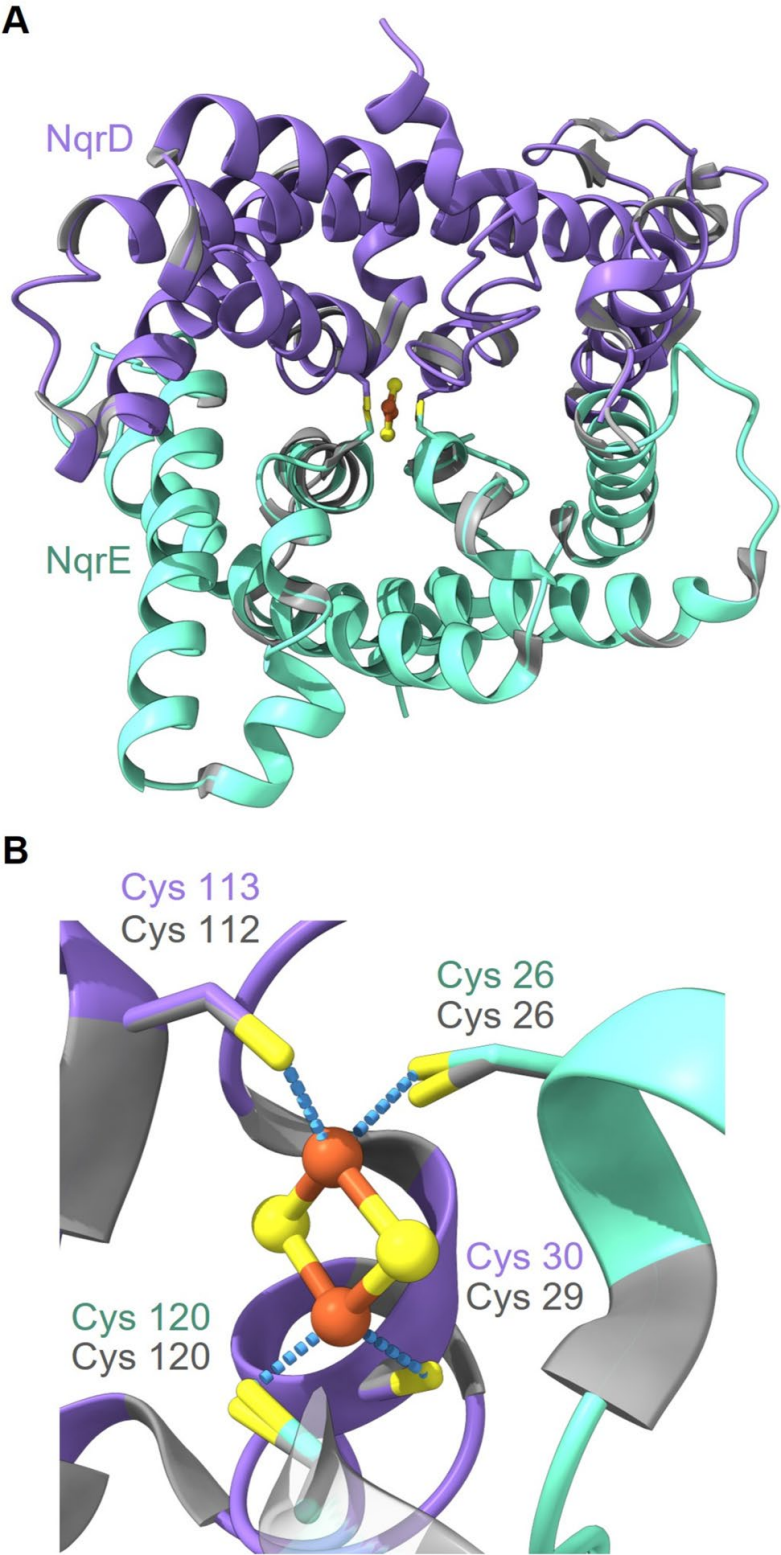
Structural alignment of the models of NqrD (purple) and NqrE (cyan) subunits from *P. bryantii* with the structures of the corresponding NqrD and NqrE subunits from *V. cholerae* (gray). **A** Subunits NqrD and NqrE each provide two cysteine residues for coordination of a [2Fe–2S] located at the interface of the two subunits. **B** Close-up view of the [2Fe–2S] cluster. The thiol groups (yellow) of the four cysteines (Cys29, Cys112 in Vc-NqrD and Cys26, Cys120 in Vc-NqrE; Cys30, Cys113 in Pb-NqrD and Cys26, Cys120 in Pb-NqrE) coordinate the [2Fe–2S] cluster (orange and yellow spheres). The coordinates of the Vc-NqrD and NqrE structures were obtained from the protein data bank (PDB 8A1W), and the Pb-NqrD and NqrE structures were modeled with Phyre2. The structures were aligned and drawn with UCFS ChimeraX version 1.5

In the integral membrane subunits Vc-NqrD and Vc-NqrE, an [2Fe–2S] cluster is coordinated by cysteine residues originating from both subunits (Cys29 and Cys112 from NqrD; Cys26 and Cys120 from NqrE) (Fig. 9A). This [2Fe–2S] is crucial for electron transfer from NqrF to NqrC across the membrane and is proposed to drive the conformational changes in Vc-NQR (Hau et al. 2023). The 3D model of Pb-NqrD and Pb-NqrE structure confirms that in Pb-NQR, the conserved corresponding four Cys residues (Cys30 and Cys113 from NqrD and Cys26 and Cys120 from NqrE) can coordinate as well a [2Fe–2S] cluster in a highly similar manner (Fig. 9B and C).

**Fig. 10:**
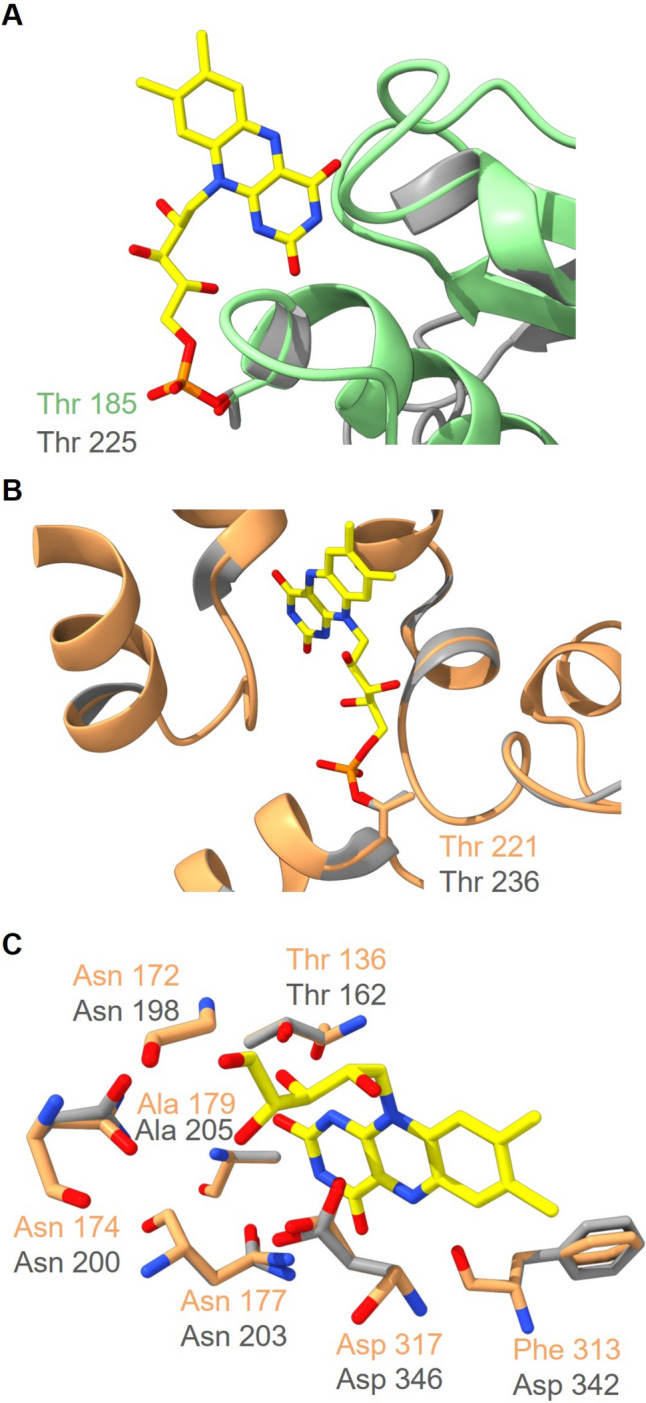
Structural alignments of the models of the NqrB (orange) and NqrC (green) subunits from *P. bryantii* with the structures of the corresponding NqrB and NqrC subunits from *V. cholerae* (gray). **A** Close-up view of the FMN binding site in NqrC with Thr225 (Vc-NqrC) and Thr185 (Pb-NqrC). **B** Close-up view of the FMN binding site in NqrB with Thr236 (Vc-NqrB) and Thr221 (Pb-NqrB). **C** Close-up view of the riboflavin-binding site in NqrB with Gly198, Asn200, Thr162, Ala205, Asn203, Phe342 and Asp346 as interaction partners of riboflavin in Vc-NqrB, and Gly172, Asn174, Thr136, Ala179, Asn177, Phe313 and Asp317 in Pb-NqrB. The coordinates of the Vc-NqrB and NqrC structures were obtained from the protein data bank (PDB 8A1W), and the Pb-NqrB and NqrC structures were modeled with Phyre2. The structures were aligned and drawn with UCFS ChimeraX version 1.5

From this membrane-bond [2Fe–2S] cluster, electrons are transferred to covalently bound FMN in NqrC. In Vc-NqrC, FMN is covalently attached to Thr225 via a phosphodiester bond (Fig. 10A) (Barquera et al. 2001; Casutt et al. 2012). In Pb-NqrC, the corresponding conserved threonine (Thr185) resides in a similar position (Fig. 10A) putting forward that FMN is covalently attached to this residue. From NqrC, the electron transfer proceeds to subunit NqrB. Like in NqrC, an FMN is covalently attached to a threonine residue. In Vc-NqrB, FMN is bound via a phosphodiester bond to threonine 236 (Fig. 10B) (Barquera et al. 2001; Casutt et al. 2012). The corresponding conserved Thr221 in Pb-NqrB is predicted in a similar position in the 3D model and could bind as well an FMN (Fig. 10B). Thus, the 3D models strongly support the previous finding that Pb-NqrC and Pb-NqrB contain covalently linked flavins (Schleicher et al. 2021a).

In Vc-NQR, electron transport from FMN in NqrB to quinone proceeds via riboflavin (Barquera et al. 2002; Juárez et al. 2008; Tao et al. 2008). Recently published 3D structures of Vc-NQR revealed the position of the riboflavin in Vc-NqrB (Kishikawa et al. 2022; Hau et al. 2023), which forms several hydrogen bonds to protein residues. The amino acid residues Gly198, Asn200, Thr162 and Asp346 form hydrogen bonds with the ribityl chain, and Ala205, Phe342, Thr162 and Asn203 interact with the isoalloxazine structure (Fig. 10C). In the predicted model of Pb-NqrB, the corresponding residues Gly172, Asn174, Thr136, Ala179, Asn177, Phe313 and Asp317 reside in very similar positions forming a riboflavin-binding site (Fig. 10C).

### Quinone reduction and Na^+^ translocation in NqrB of Pb-NQR

**Fig. 11:**
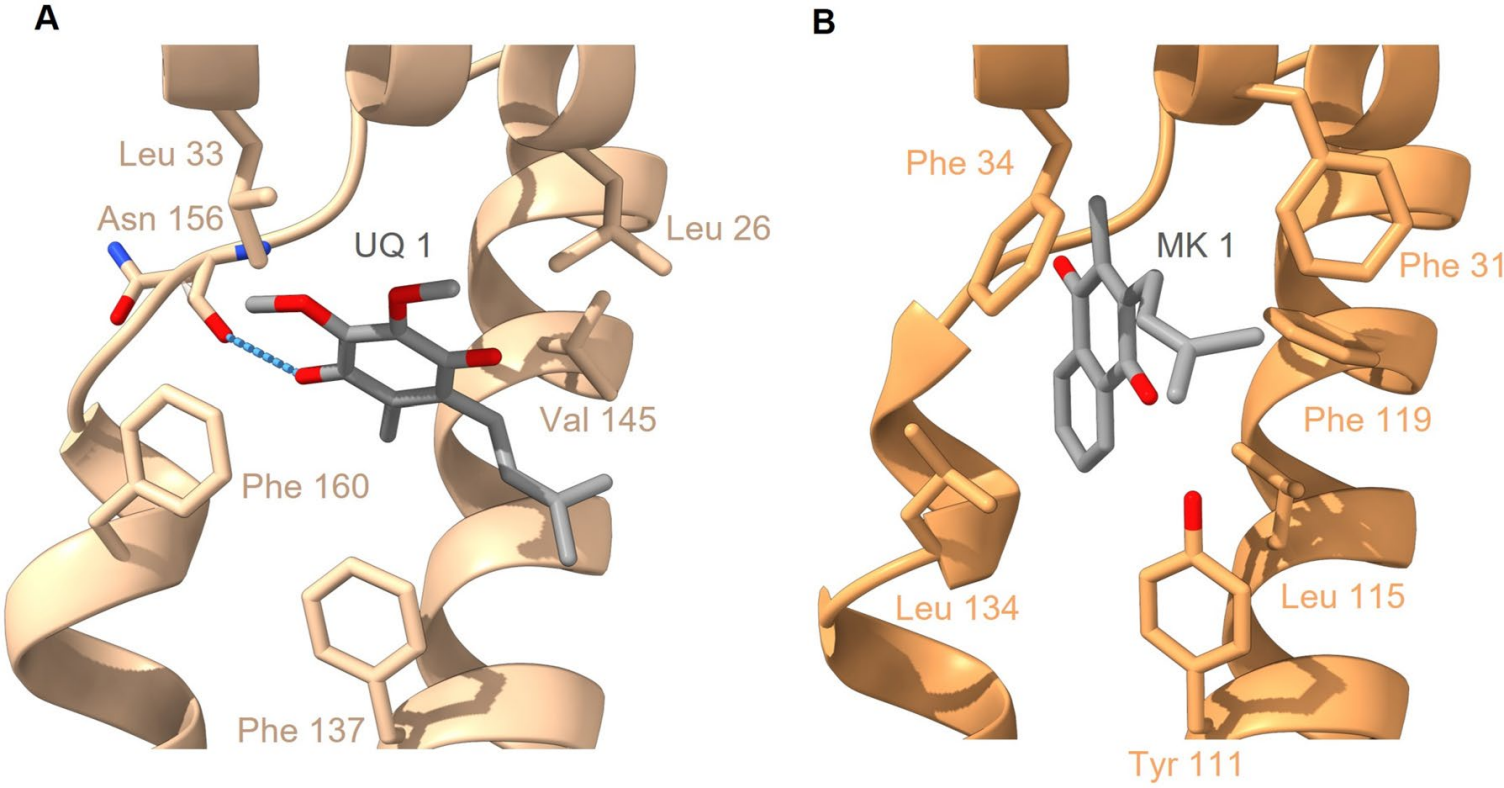
Predicted mode of menaquinone binding to NQR from *P. bryantii*. **A** Experimentally determined structure of ubiquinone-1 bound to subunit NqrB of Vc-NQR (PDB: 8A1W). Residues Leu26, Leu33, Phe137, Val145 and Phe160 forming the binding site are indicated. **B** Structural model of the quinone binding site in Pb-NqrB characterized by residues Phe31, Phe34, Tyr111, Leu115, Phe119 and Leu134. Menaquinone was docked into the modeled structure using OpenEye docking suite (4.1.2.). Carbon atoms of quinones are shown in gray and oxygen atoms in red. Structures were drawn with UCFS ChimeraX version 1.5

From the riboflavin in NqrB, electrons are transferred further to quinone, which serves as an electron shuttle between enzymes of the respiratory chain. The structures of Vc-NQR in complex with UQ-1 or UQ-2 provided first insights into the binding site of UQ in subunit NqrB (Hau et al. 2023). At a rim of NqrB, UQ-1/UQ-2 binds with its head group close to the cytoplasmic aspect of the membrane (Fig. 11A). The interaction of UQ-1/UQ-2 with NqrB is promoted by two amphiphilic helices at the N-terminus of NqrB. The residues Leu26, Leu33, Phe137, Val145 and Phe160 form a hydrophobic pocket for UQ. Furthermore, the carboxyl of the headgroup of UQ forms a hydrogen bond to Asn156. In the model of Pb-NqrB, also two N-terminal amphiphilic helices are predicted (Fig. 11B) and a hydrophobic pocket is formed by the amino acid residues Phe31, Phe34, Tyr111, Phe119 and Leu134 (Fig. 11B). This predicted quinone site in Pb-NQR differs from the site in Vc-NqrB. This difference reflects that Pb-NQR utilizes menaquinone instead of ubiquinone, as *P. bryantii* exclusively relies on menaquinone for respiration (Schleicher et al. 2021a). To characterize the putative mode of menaquinone binding in Pb-NQR, we performed docking calculations. The results of the docking calculations revealed a binding mode for menaquinone-1, which deviates significantly from UQ binding in Vc-NqrB. In the structure of Vc-NqrB, the headgroup of UQ-2 is tilted by about 45° with respect to the transmembrane helices and the isoprenoid tail of UQ-2 is in van-der-Waals with the proximate transmembrane helix of NqrB. Important for this interaction in Vc-NqrB is residue Gly141 in the transmembrane helix. Gly lacks a sidechain and therefore allows the close interaction of the isoprenoid tail. In contrast, in Pb-NqrB, Leu115 with a bulky sidechain resides in this position. The results of the docking calculations for menaquinone-1 suggest that the naphthoquinone and the isoprenoid moiety of menaquinone-1 interact differently with Pb-NqrB. The naphthoquinone ring is oriented parallel to the transmembrane helices and the isoprenoid moiety exhibits a different orientation. Thus, the 3D model of Pb-NqrB provides some details for the different quinone specificity of NQR from both species.

In Vc-NQR, NqrB exhibits a Na^+^ translocating pathway that is lined by the backbone carbonyls of residues Val161, Ile164, Leu168 and a constriction formed by the sidechains of Phe338 and Phe342 (Steuber et al. 2014; Hau et al. 2023). In Pb-NqrB, the corresponding residues are strictly conserved and residues Val135, Ile138, Leu142, Phe309, Phe313 reside at the same positions as observed in Vc-NqrB (Steuber et al. 2014; Hau et al. 2023). This strongly suggests that the pathway for Na^+^ translocation in Pb-NQR is highly similar to Vc-NQR.

### Occurrence of QFR and NQR in representatives from bacterial phyla

Succinate:quinone reductases (SQR) are divided into the four types A–D based on their architecture (number of subunits and heme *b* content). Type A harbors two hydrophobic subunits with two hemes *b* (Hägerhäll and Hederstedt 1996). Type A enzymes oxidize succinate, using methylmenaquinone (thermoplasmaquinone) as electron acceptor, and operate as succinate:quinone reductases (SQR) in vivo. SQRs belonging to type B harbor three subunits, containing two hemes *b*. This type of enzyme is known to operate in both directions with the MK/MKH_2_ pool as electron acceptor/donor (Hägerhäll 1997). SQRs belonging to type C, with two membranous subunits and one heme *b* (Yankovskaya et al. 2003), also operate in both directions, but exclusively with high potential quinones, such as ubiquinone (UQ) (Hägerhäll and Hederstedt 1996). Type D enzymes represent QFRs with two hydrophobic subunits, which lack heme cofactors (Hägerhäll and Hederstedt 1996; Iverson et al. 1999).

The four types of SQRs can also be categorized into three functional classes, based on their quinone specificity and their in vivo function (Lancaster 2004). Subclass 1 represents SQRs, oxidizing succinate and reducing high potential quinones, such as UQ. QFRs couple fumarate reduction to the oxidation of a low-potential quinol and belong to subclass 2. Enzymes belonging to subclass 3 represent SQRs, which oxidize succinate and reduce low-potential quinones (Lancaster 2004). Note that enzymes of subclass 1 and 2 catalyze exergonic reactions, while the reaction of subclass 3 enzymes is endergonic (under standard conditions at pH 7.0).

Members of different types and classes of the SQR family are broadly distributed in the bacterial kingdom, reflecting their high variability in terms of substrate specificity and modes of energy conversion (Unden and Dünnwald 2008). We searched for different SQR types in representatives from bacterial phyla (supplementary material table S1). QFRs oxidizing low-potential quinols, which are composed of three subunits and contain two hemes b (type B), are present in the phyla Chlorobiota, Bacteroidota, Actinomycetota, Thermodesulfobacteriota and Campylobacterota. Typical examples are QFR from *Desulfovibrio gigas*, a member of the Deltaproteobacteria (phylum Thermodesulfobacteriota), and *Wolinella succinogenes*, a member of the Epsilonproteobacteria (phylum Campylobacterota). SQRs oxidizing succinate and reducing high potential quinones are present in Alpha-, Beta- and Gammaproteobacteria. Some representatives from the Gammaproteobacteria comprise both UQ and MK, enabling the organism to utilize either succinate or fumarate prevalent under different environmental conditions with adequate catalytic pathways. Consequently, these organisms encode both SQR and QFR, which are expressed differentially. Typically, their QFRs are type D enzymes (harboring two hydrophobic subunits but no heme), oxidizing low-potential quinols and reducing fumarate (Hägerhäll 1997). Bacteria belonging to Bacillota operate SQRs, which oxidize succinate and reduce low-potential quinones in a reaction that is endergonic under standard conditions at pH 7.0.

*Prevotella bryantii* and *Prevotella bivia* belong to the family *Prevotellaceae*, which is assigned to the phylum Bacteroidota (Avgustin et al. 1997). These obligate anaerobes contain MK as the only reported quinone species (Schleicher et al. 2021a). They comprise a type B QFR with three subunits and two hemes *b*, which preferentially oxidizes menaquinol (MKH_2_) under reduction of fumarate in vivo.

Selected organisms of the phylum Bacteroidota (*Bacteroides fragilis, Prevotella bryantii, Prevotella bivia*) and members of the Gammaproteobacteria (*Haemophilus influenzae*) contain both NQR and QFR (supplementary table S1 and table S2). It seems likely that *B. fragilis* and *H. influenzae* may utilize both enzymes to re-oxidize NADH concomitant with the build-up of an electrochemical membrane potential as shown for *P. bryantii* (Schleicher et al. 2021a) and *P. bivia* (Schleicher et al. 2021b).

## Conclusion

The NQR and QFR represent the key enzymes of energy conservation in *P. bryantii*. Here, the reduction of fumarate to succinate using NADH as electron donor is coupled to chemiosmotic energy conservation by SMF generation with menaquinone as redox carrier. From 3D structural models of Pb-NQR and Pb-QFR, routes for electron transfer and substrate binding sites are predicted. This mode of energy conservation is not only important for *P. bryantii,* but is likely to occur in other bacteria operating both NQR and QFR.

### Supplementary Information

Below is the link to the electronic supplementary material.

Supplementary file1 (DOCX 877 KB)

## Abbreviations

NQR: Na^+^-translocating NADH:quinone oxidoreductase
QFR: Quinol:fumarate reductase
SQR: Succinate:quinone reductase
FNO: Ferredoxin:NAD^+^ oxidoreductase
RNF: Rhodobacter nitrogen fixation complex
MK: Menaquinone
UQ: Ubiquinone
