# Supplementary material for: Functionality of the Na+-translocating NADH:quinone oxidoreductase and quinol:fumarate reductase from Prevotella bryantii inferred from homology modeling
Source: Arch Microbiol. 2023 Dec 21;206(1):32. doi: 10.1007/s00203-023-03769-5 (PMC10739449; doi:10.1007/s00203-023-03769-5)
Supplement: Supplementary file 1 — Supplementary file1 (DOCX 877 KB) [file 203_2023_3769_MOESM1_ESM.docx]

Supplementary Material

**Functionality of the Na^+^-translocating NADH:quinone oxidoreductase and quinol:fumarate reductase from *Prevotella bryantii* inferred from homology modeling**

Jann-Louis Hau^1,#^, Lena Schleicher^1,2,#^, Sebastian Herdan^1,2^, Jörg Simon^3^, Jana Seifert^2,4^, Günter Fritz^1^, Julia Steuber^1,2,*^

^1^Institute of Biology, University of Hohenheim, Garbenstraße 30, 70599 Stuttgart, Germany

^2^HoLMiR- Hohenheim Center for Livestock Microbiome Research, University of Hohenheim, Leonore-Blosser-Reisen-Weg 3, 70599 Stuttgart, Germany

^3^Microbial Energy Conservation and Biotechnology, Department of Biology, Technical University of Darmstadt, Schnittspahnstraße 10, 64287 Darmstadt, Germany

^4^Institute of Animal Science, University of Hohenheim, Emil-Wolff-Straße 8, 70599 Stuttgart, Germany

*Correspondence: Julia Steuber, [julia.steuber@uni-hohenheim.de](mailto:julia.steuber@uni-hohenheim.de)

^#^These authors contributed equally.

**Protein homology modelling**

Structural models of the *P. bryantii* NQR (Pb-NQR) and QFR (Pb-QFR) were generated based on the 3D structures of the highly related enzymes from *Vibrio cholerae* (PDB 8A1W, PDB 8ACY) (Hau et al. 2023) and *Desulfovibrio gigas* (PDB 5XMJ) (Guan et al. 2018)*.* Amino acid sequences of Pb-NQR and Pb-QFR subunits (queries) were retrieved from Uniprot. Structural models of Pb-QFR were generated using Phyre2 (Kelley et al. 2015) and AlphaFold (Jumper et al. 2021), and the resulting models were compared. Subunits of the proten complexes were modelled individually and were superimposed on the template structures. Phyre2 uses an experimentally determined structure of a homolog as a template to predict the structure by performing Hidden-Markov-model(HMM)-HMM matching. This allows for modelling of a protein in the conformation of the used template. AlphaFold performs structure predictions utilizing neural networks trained by the evolutionary and physical constraints derived from known protein structures. At first the neural network creates an array that represents a processed multiple sequence alignment. In the neural network block called Evoformer a structural hypothesis is formed and refined. This procedure is followed by a structure module that builds a definite preliminary structure by rotating and translating each residue of the protein. This initial structure is refined allowing simultaneous local refinement of all parts of the structure. The refinement of the structure is an iterative process feeding the outputs of the modules recursively into the same modules yielding highly accurate structures. Note that the Pb-NQR model generated by AlphaFold represents a conformation as observed in the X-ray structure of Vc-NQR (8ACY). Phyre2 uses an experimentally determined structure of a homolog as a template to predict the structure by performing Hidden-Markov-model(HMM)-HMM matching. This allows for modelling of a protein in the conformation of the respective template. Pb-QFR was modelled in the “normal mode” of Phyre2 using structural templates available on the PHYRE2 protein fold recognition server. Pb-NQR was modelled in the “One-to-One threading” mode of Phyre2 using structures of Vc-NQR in different conformations from the Protein Data Bank (PDB 8A1W, PDB 8ACY). In Phyre2 four consecutive core methods are used for homology modelling (Kelley et al. 2015). In the first stage, the query amino acid sequence is scanned against a sequence database by the software HHblits, resulting in an evolutionary sequence profile. Furthermore, a secondary structure of the protein is predicted with the software PSPI-Pred16 (Kelley et al. 2015). These results are used in the second stage by Phyre2 to create a hidden Markov model (HMM). The HMM is a stochastic model, describing different states of a system and the probabilities of their transitions. In this case, the HMM invokes different states (positions) of the amino acids in the structure of the query protein. Every state has an occurrence probability and a transition probability, which indicates the probability of moving from one state to another (Eddy 2004). Phyre2 scans the generated HMM against a pre-compiled database (fold library) of HMMs from proteins with an experimentally determined protein structure, resulting in a list of structural based query-template alignments (HMM-HMM matching). This matching process allows the prediction of significant protein sequence similarities and their positions in the modelled structure, including insertions and deletions along the alignment (Söding 2005). HMM-HMM matching is also used to predict secondary structure types and transmembrane regions (Bystroff and Krogh 2008). Consequently, these alignments are the basis to create crude backbone models. In the third stage, a library of fragments and the (CCD)17 algorithm are used to correct insertions or deletions in the initial crude model. Finally, sidechain fitting to the backbone is performed using R3 protocol20 (Kelley et al. 2015). For every model, different factors (confidence value, coverage, sequence identity, TM score) are calculated allowing estimation of the quality of the model. These calculations are based on the alignments of the HMM-HMM matchings and thus, not only the amino acid sequences are taken into consideration, but also the amino acid positioning in the 3D model (Söding 2005). The coverage value gives the percentage of coverage between the query and the template. The sequence identity is the proportion of the protein residues, which correspond to identical template residues in the generated alignment (Kelley et al. 2015). With a structure derived sequence identity > 30%, the model has a high accuracy, and the side chain conformations of conserved residues exhibit the same orientations. The confidence value represents the probability (from 0 to 100%) that the query and the template are homologous (Kelley et al. 2015), but it is no direct measure for the accuracy of the model. However, a model with > 90% confidence indicates that the query structure is very likely to adopt to the overall fold of the template structure. Moreover, it suggests that the core of the protein is modelled at high accuracy. The template modelling (TM) score (from 0-1) is a measure of similarity between the modelled protein and the template structure (Kelley et al. 2015). Identical structures score 1.0, whereas a score < 0.2 indicates a similarity no better than random. Next to these basic information of the modelled structure quality, Phyre2 gives more detailed information about the secondary structure and the different domains. Here, the ProQ2 assessment, clash analysis, rotamer analysis, Ramachandran analysis and the alignment confidence are shown. The results of these analyses are all presented in color-coded structures of the modelled protein, allowing local qualification of the structure. ProQ2 is a model quality assessment algorithm, predicting and visualizing local as well as global structural model correctness (color code: from red, good, to blue, bad) (Ray et al. 2012). By clash analysis, atoms in residues are identified, which may lie to close to one another in the model. A large number of clashes could indicate bad sidechain placement or an incorrect backbone in this region (color code: from blue, good, to red, bad). The Rotamer analysis shows side chains, which may have not been modelled ideally (stage 4 of protein modelling by Phyre2; color code: blue, good; red, bad). The Ramachandran analysis asks, if residues in the model lie in electrostatically favorable, allowed or disallowed regions. Thus, it addresses the stereochemistry and geometry of the modelled structure (color code: blue, good; green, allowed; red, bad). The alignment confidence shows the reliability of the pairwise query-template alignment of the HMM-HMM matching (color code: from red, good, to blue, bad).

**Table S1** SQRs and QFRs of bacteria from the phyla Bacillota, Actinomycetota, Bacteroidota, Chlorobiota and Proteobacteria. Enzymes were detected under specified growth conditions (aerobic or anaerobic) described in the corresponding studies cited here. The proposed directionality of the enzymatic reaction (succinate oxidation, SQR; fumarate reduction, QFR) is indicated. Subunits (SU) are listed with Uniprot accession numbers and calculated molecular weights, as well as the heme content in subunits C and D. Furthermore, the categorization of the SQRs into the functional subclasses (1-3) is indicated. The dominant quinone species at the indicated growth condition is also listed. Subunits marked with “?” are present as shown by experimental data, but no hit in the Uniprot data base could be found. This could be due to erroneous annotation, or incomplete genome information. Subunits of *C. mediatlanticus* TB-2 do not have Uniport accession numbers. NCBI nomenclature is used instead. Presence of the NQR complex in these strains is indicated with + (present) or – (not present). *B. subtilis, Bacillus subtilis; A. viscosus, Actinomyces viscosus; B. fragilis, Bacteroides fragilis; C. marinum, Cyclobacterium marinum; F. canadensis, Flexibacter canadensis; F. succinicans, Flavobacterium succinicans; P. bryantii, Prevotella bryantii; P. bivia, Prevotella bivia; R. marinus, Rhodothermus marinus; C. jejuni, Campylobacter jejuni; C. mediatlanticus, Caminibacter mediatlanticus; D. gigas, Desulfovibrio gigas; E. coli, Escherichia coli; G. sulfurreducens, Geobacter sulfurreducens; H. influenzea, Haemophilus influenzae; H. pylori, Helicobacter pylori; N. meningitidis, Neisseria meningitidis; P. aeruginosa, Pseudomonas aeruginosa; P. denitrificans, Paracoccus denitrificans; V. cholerae, Vibrio cholerae; W. succinogenes, Wolinella succinogenes*

| **phyla/organisms** | **specific**  **growth condition** | **proposed reaction *in vivo***  **(SQR or QFR)** | **SU A** | **SU B** | **SU C** | **SU D** | **number of hemes *b*** | **subclass** | **quinone type** | **references** | **NQR** |  |
| --- | --- | --- | --- | --- | --- | --- | --- | --- | --- | --- | --- | --- |
| **Bacillota** |  |  |  |  |  |  |  |  |  |  |  |  |
| *B. subtilis*  strain 168 | aerobic | SQR | P08065  65 kDa | P08066  28 kDa | P08064  23 kDa | - | 2 | 3 | MK | (Hägerhäll et al. 1992) | - |  |
| **Actinomycetota** |  |  |  |  |  |  |  |  |  |  |  |  |
| *A. viscosus* C505 | anaerobic | QFR | F2V166  75 kDa | F2V167  28 kDa | F2V165  28 kDa | - | 2 | 2 | MK | (Takahashi et al. 1994) | - |  |
| **Bacteroidota** |  |  |  |  |  |  |  |  |  |  |  |  |
| *B. fragilis* | anaerobic | QFR | Q7X479  72 kDa | Q7X478  27 kDa | Q7X480  26 kDa | - | 2 | 2 | MK | (Baughn and Malamy 2003) | + |  |
| *F. succinicans* | anaerobic | QFR | A0A199XQQ8  74 kDa | A0A199XPJ3  28 kDa | A0A199XPU1  25 kDa | - | 2 | 2 | MK | (Callies and Mannheim 1978) | - |  |
| *P. bryantii* B_1_4 | anaerobic | QFR | D8DXM6  73 kDa | D8DXM5  28 kDa | D8DXM7  26 kDa | - | 2 | 2 | MK | (Schleicher et al. 2021a) | + |  |
| *P. bivia* DSM 20514 | anaerobic | QFR | I4Z8D9  73 kDa | I4Z8E0  27 kDa | I4Z8D8  25 kDa | - | 2 | 2 | MK | (Schleicher et al. 2021b) | + |  |
| **Rhodothermota** |  |  |  |  |  |  |  |  |  |  |  |  |
| *R. marinus*  ATCC 43812 | aerobic | SQR | D0MD06  65 kDa | D0MD07  27 kDa | D0MD05  19 kDa | - | 2 | 3 | MK | (Fernandes et al. 2001) | - |  |
| **Thermodesulfo-bacteriota**  *G. sulfurreducens* ATCC 51573 | | anaerobic | QFR | Q74DY8  71 kDa | Q74DY7  28 kDa | Q74DY9  24 kDa | - | 2 | 2 | MK | (Butler et al. 2006) | - |
| *D. gigas*  ATCC 19364 | | anaerobic | QFR | T2GB49  69 kDa | T2G9X8  30 kDa | T2GAT5  25 kDa | - | 2 | 2 | MK | (Guan et al. 2018) | - |
| **Campylobacterota**  *C. jejuni*  ATCC 700819 | | anaerobic | QFR | Q0PBA1  74 kDa | Q0PBA0  28 kDa | Q0PBA2  30 kDa | - | 2 | 2 | MK | (Weingarten et al. 2009) | - |
| *H. pylori*  ATCC 700824 | | anaerobic | QFR | Q9ZMP0  80 kDa | Q9ZMP1  28 kDa | Q9ZMN9  29 kDa | - | 2 | 2 | MK | (Ge et al. 2000) | **-** |
| *W. succinogenes* ATCC 29543 | | anaerobic | QFR | P17412  73 kDa | P17596  27 kDa | P17413  30 kDa | - | 2 | 2 | MK | (Lancaster et al. 1999) | - |
| *C. mediatlanticus* TB-2 | | anaerobic | QFR | WP_138323656.1  63 kDa | WP_007473051  37 kDa | ? | - | 2 | 2 | MK | (Vetriani et al. 2014) | - |
| **Pseudomonadota** |  |  |  |  |  |  |  |  |  |  |  |  |
| *E. coli* K12 | | anaerobic | QFR | P00363  66 kDa | P0AC47  27 kDa | P0A8Q0  15 kDa | P0A8Q3  13 kDa | - | 2 | MK | (Cecchini et al. 2002) | - |
| *E. coli* K12 | aerobic | SQR | P0AC41  64 kDa | P07014  27 kDa | P69054  14 kDa | P0AC44  13 kDa | 1 | 1 | UQ | (Cecchini et al. 2002) | - |  |
| *H. influenzae*  ATCC 51907 | | anaerobic | QFR | P44894  66 kDa | P44893  29 kDa | P44892  15 kDa | P44891  13 kDa | - | 2 | MK | (Hägerhäll 1997) | + |
| *H. influenzae* ATCC 51907 | aerobic | SQR | ? | ? | ? | ? | 1 | 1 | UQ | (Othman et al. 2014) | + |  |
| *N. meningitidis* MC58 | aerobic | SQR | Q9JZP8  65 kDa | Q7DDK2  27 kDa | Q7DDK3  14 kDa | Q9JZP9  13 kDa | 1 | 1 | UQ | (Clark et al. 1987) | **+** |  |
| *P. aeruginosa* ATCC 15692 | aerobic | SQR | Q9I3D5  64 kDa | Q9I3D4  26 kDa | Q9I3D7  14 kDa | Q9I3D6  14 kDa | 1 | 1 | UQ | (Liang et al. 2020) | **+** |  |
| *P. denitrificans* | aerobic | SQR | Q59661  66 kDa | Q59662  30 kDa | Q59659  14 kDa | Q59660  14 kDa | 1 | 1 | UQ | (Hederstedt 2002) | **-** |  |
| *V. cholerae*  M66-2 | aerobic | SQR | C3LS85  66 kDa | C3LS86  28 kDa | C3LS87  15 kDa | C3LS88  14 kDa | 1 | 1 | UQ | (Bueno et al. 2020) | + |  |

**Table S2** Identification of electrogenic NADH:quinone oxidoreductases (NQR) in representatives of bacterial phyla operating a quinol:fumarate reductase (QFR) utilizing menaquinone (B type). Four bacterial species were identified which contain both QFR (B-type) and NQR. To verify the presence of the NQR, amino acid sequences of subunits from the Na^+^ -translocating NQR from *Vibrio cholerae* serotype O1 (strain ATCC 39541) were used to search for the corresponding subunits in the genomes of the selected bacterial species using BLAST. Uniport accession numbers of the template NQR subunits are given, as well as the percent identities resulting from the BLAST searches. *B. fragilis, Bacteroides fragilis; P. bryantii, Prevotella bryantii; P. bivia, Prevotella bivia, H. influenzae, Haemophilus influenzae*

| Template protein | *B. fragilis*  3397 T10*** | *P. bryantii*  b_1_4* | *P. bivia*  DSM 20514 | *H. influenzae*  ATCC51907* |
| --- | --- | --- | --- | --- |
| NqrA  (A5F5X1) | 36 % | 32 % | 32 % | 63 % |
| NqrB  (A5F5X0) | 41 % | 40 % | 39 % | 71 % |
| NqrC  (A5F5Y7) | 29 % | 27 % | 38 % | 53 % |
| NqrD  (A5F5Y6) | 76 % | 55 % | 54 % | 76 % |
| NqrE  (A5F5Y5) | 60 % | 58 % | 57 % | 82 % |
| NqrF  (A5F5Y4) | 51 % | 47 % | 46 % | 82 % |
| RnfB  (P77223) | 33 % | 36 % | -- | 88 % |

*Organisms containing next to a *nqr* operon also a predicted *rnf* operon. This was analyzed by using the amino acid sequence of RnfB from *Escherichia coli* K12 (Uniprot accession number P77223) as template for a genome search with BLAST. Percent identities to the identified RnfB homolog are given in the table.

**Table S3** Quality of the 3D models of subunits of Pb-QFR. Homology modelling of the QFR subunits from *P. bryantii* was performed with Phyre2 in the “normal mode”. Given are the templates for modelling, which were selected by Phyre2, as well as the confidence, coverage, identity and template modelling (TM) score.

|  | | **template** | **Confidence [%]** | **Coverage [%]** | **Identity [%]** | **TM score** |
| --- | --- | --- | --- | --- | --- | --- |
| **QFR** | FrdA | FrdA *D. gigas*  (PDB 5XMJ) | 100 | 95 | 28 | 1.0 |
|  | FrdB | FrdB *D. gigas*  (PDB 5XMJ) | 100 | 98 | 23 | 1.0 |
|  | FrdC | FrdC *D. gigas*  (PDB 5XMJ) | 100 | 86 | 17 | 1.0 |

**Table S4** Quality of the 3D models of subunits of Pb-NQR. Homology modelling was performed with Phyre2 in the “One-to-one threading mode” based on the structures of subunits of Vc-NQR. Templates used for modelling are indicated.

|  | | **template** | **Confidence [%]** | **Coverage [%]** | **Identity [%]** |
| --- | --- | --- | --- | --- | --- |
| **NQR** | NqrA | NqrA *V. cholerae*  (PDB 8A1W) | 100 | 94 | 32 |
|  | NqrB | NqrB *V. cholerae*  (PDB 8A1W) | 100 | 89 | 46 |
|  | NqrC | NqrC *V. cholerae*  (PDB 8A1W) | 100 | 79 | 29 |
|  | NqrD | NqrD *V. cholerae*  (PDB 8A1W) | 100 | 95 | 53 |
|  | NqrE | NqrE *V. cholerae*  (PDB 8A1W) | 100 | 97 | 59 |
|  | NqrF | NqrF *V. cholerae*  (PDB 8A1W) | 100 | 98 | 48 |
|  |  |  |  |  |  |
|  | NqrA | NqrA *V. cholerae*  (PDB 8ACY) | 100 | 85 | 32 |
|  | NqrB | NqrB *V. cholerae*  (PDB 8ACY) | 100 | 81 | 46 |
|  | NqrC | NqrC *V. cholerae*  (PDB 8ACY) | 100 | 79 | 29 |
|  | NqrD | NqrD *V. cholerae*  (PDB 8ACY) | 100 | 96 | 52 |
|  | NqrE | NqrE *V. cholerae*  (PDB 8ACY) | 100 | 98 | 59 |
|  | NqrF | NqrF *V. cholerae*  (PDB 8ACY) | 100 | 98 | 48 |


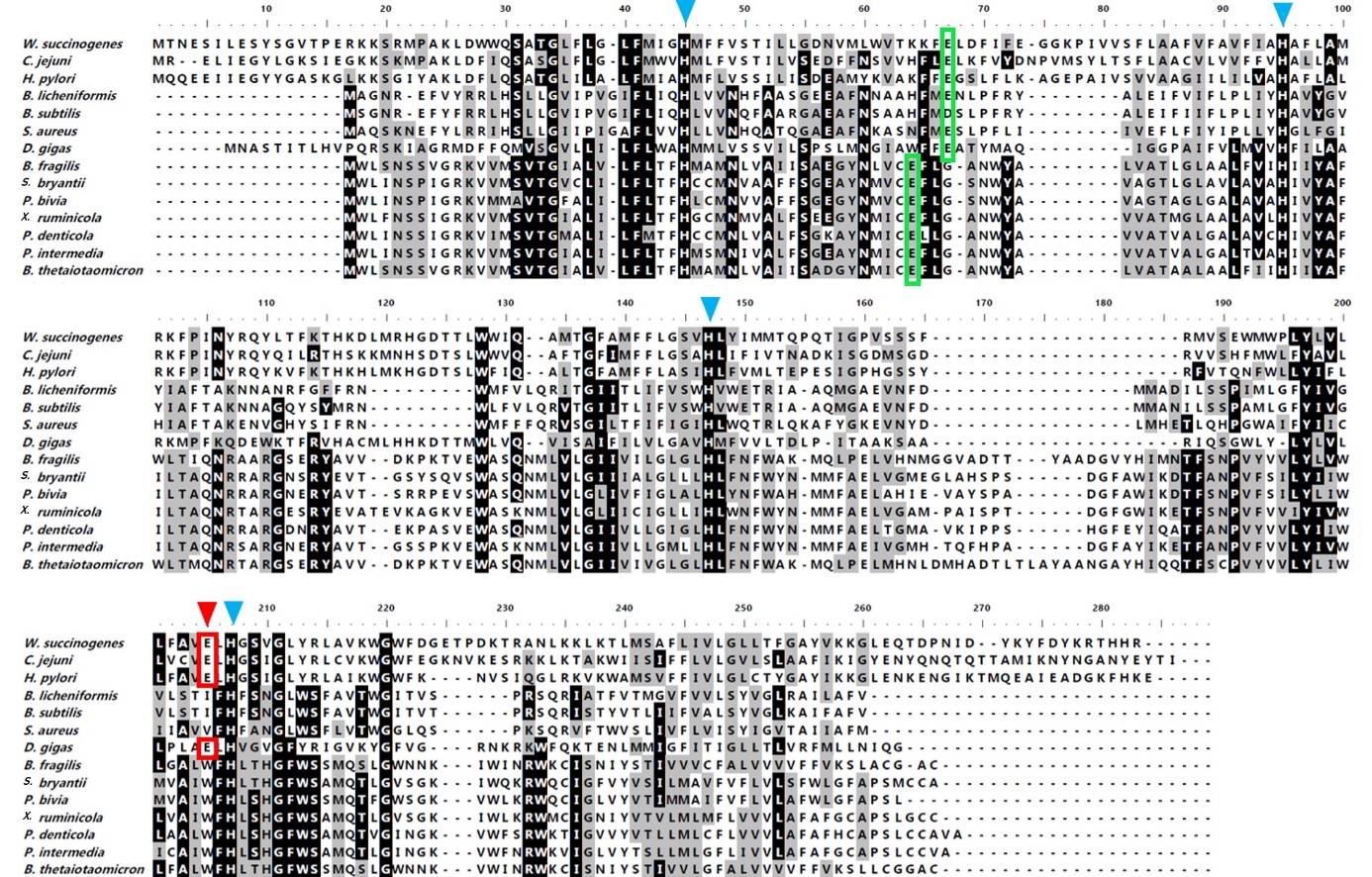


**Fig. S1** Sequence alignment of the C- subunits of diheme-containing succinate:menaquinone oxidoreductases from different bacterial species. Gram-negative bacteria: Epsilonproteobacteria (*W. succinogenes, C. jejuni, H. pylori*), Betaproteobacteria (*D. gigas*); Bacteroidota (*B. fragilis, P. bryantii, P. bivia, X. ruminicola, P. denticola, P. intermedia, B. thetaiotaomicron*). Gram-positive bacteria: *B. licheniformis, B. subtilis, S. aureus*. Identical amino acid residues (black) and similar amino acid residues (grey) are highlighted. The highly conserved His ligands of the heme groups are indicated by blue triangles. The Glu residue in the putative menaquinol oxidation site is highlighted in green. The proposed essential amino acid residue for the E-pathway is indicated by a red triangle (highlighted in red).

References

Baughn AD, Malamy MH (2003) The essential role of fumarate reductase in haem-dependent growth stimulation of *Bacteroides fragilis*. Microbiology 149:1551–1558. https://doi.org/10.1099/mic.0.26247-0

Bueno E, Pinedo V, Cava F (2020) Adaptation of *Vibrio cholerae* to hypoxic environments. Front Microbiol 11:739. https://doi.org/10.3389/fmicb.2020.00739

Butler JE, Glaven RH, Esteve-Núñez A, Núñez C, Shelobolina ES, Bond DR, Lovley DR (2006) Genetic characterization of a single bifunctional enzyme for fumarate reduction and succinate oxidation in *Geobacter sulfurreducens* and engineering of fumarate reduction in *Geobacter metallireducens*. J Bacteriol 188:450–455. https://doi.org/10.1128/JB.188.2.450-455.2006

Bystroff C, Krogh A (2008) Hidden Markov Models for prediction of protein features. Methods Mol Biol 413:173–198. https://doi.org/10.1007/978-1-59745-574-9_7

Callies E, Mannheim W (1978) Classification of the Flavobacterium-Cytophaga complex on the basis of respiratory quinones and fumarate respiration. Int J Sys Evol Microbiol 28:14–19. https://doi.org/10.1099/00207713-28-1-14

Cecchini G, Schröder I, Gunsalus RP, Maklashina E (2002) Succinate dehydrogenase and fumarate reductase from *Escherichia coli*. Biochim Biophys Acta 1553:140–157. https://doi.org/10.1016/s0005-2728(01)00238-9

Clark VL, Campbell LA, Palermo DA, Evans TM, Klimpel KW (1987) Induction and repression of outer membrane proteins by anaerobic growth of *Neisseria gonorrhoeae*. Infect Immun 55:1359–1364. https://doi.org/10.1128/iai.55.6.1359-1364.1987

Eddy SR (2004) What is a hidden Markov model? Nat Biotechnol 22:1315–1316. https://doi.org/10.1038/nbt1004-1315

Fernandes AS, Pereira MM, Teixeira M (2001) The succinate dehydrogenase from the thermohalophilic bacterium *Rhodothermus marinus:* redox-Bohr effect on heme b_L_. J Bioenerg Biomembr 33:343–352. https://doi.org/10.1023/a:1010663424846

Ge Z, Feng Y, Dangler CA, Xu S, Taylor NS, Fox JG (2000) Fumarate reductase is essential for *Helicobacter pylori* colonization of the mouse stomach. Microb Pathog 29:279–287. https://doi.org/10.1006/mpat.2000.0391

Guan H-H, Hsieh Y-C, Lin P-J, Huang Y-C, Yoshimura M, Chen L-Y, Chen S-K, Chuankhayan P, Lin C-C, Chen N-C, Nakagawa A, Chan SI, Chen C-J (2018) Structural insights into the electron/proton transfer pathways in the quinol:fumarate reductase from *Desulfovibrio gigas*. Sci Rep 8:14935. https://doi.org/10.1038/s41598-018-33193-5

Hägerhäll C, Aasa R, Wachenfeldt C von, Hederstedt L (1992) Two hemes in *Bacillus subtilis* succinate:menaquinone oxidoreductase (complex II). Biochemistry 31:7411–7421. https://doi.org/10.1021/bi00147a028.

Hägerhäll C (1997) Succinate: quinone oxidoreductases. Biochim Biophys Acta 1320:107–141. https://doi.org/10.1016/s0005-2728(97)00019-4

Hau J-L, Kaltwasser S, Muras V, Casutt MS, Vohl G, Claußen B, Steffen W, Leitner A, Bill E, Cutsail GE, DeBeer S, Vonck J, Steuber J, Fritz G (2023) Conformational coupling of redox-driven Na^+^-translocation in *Vibrio cholerae* NADH:quinone oxidoreductase. Nat Struct Mol Biol. https://doi.org/10.1038/s41594-023-01099-0

Hederstedt L (2002) Succinate:quinone oxidoreductase in the bacteria *Paracoccus denitrificans* and *Bacillus subtilis*. Biochim Biophys Acta 1553:74–83. https://doi.org/10.1016/s0005-2728(01)00231-6

Jumper J, Evans R, Pritzel A, Green T, Figurnov M, Ronneberger O, Tunyasuvunakool K, Bates R, Žídek A, Potapenko A, Bridgland A, Meyer C, Kohl SAA, Ballard AJ, Cowie A, Romera-Paredes B, Nikolov S, Jain R, Adler J, Back T, Petersen S, Reiman D, Clancy E, Zielinski M, Steinegger M, Pacholska M, Berghammer T, Bodenstein S, Silver D, Vinyals O, Senior AW, Kavukcuoglu K, Kohli P, Hassabis D (2021) Highly accurate protein structure prediction with AlphaFold. Nature 596:583–589. https://doi.org/10.1038/s41586-021-03819-2

Kelley LA, Mezulis S, Yates CM, Wass MN, Sternberg MJE (2015) The Phyre2 web portal for protein modeling, prediction and analysis. Nat Protoc 10:845–858. https://doi.org/10.1038/nprot.2015.053

Lancaster CR, Kröger A, Auer M, Michel H (1999) Structure of fumarate reductase from *Wolinella succinogenes* at 2.2 A resolution. Nature 402:377–385. https://doi.org/10.1038/46483

Liang P, Fang X, Hu Y, Yuan M, Raba DA, Ding J, Bunn DC, Sanjana K, Yang J, Rosas-Lemus M, Häse CC, Tuz K, Juárez O (2020) The aerobic respiratory chain of *Pseudomonas aeruginosa* cultured in artificial urine media: Role of NQR and terminal oxidases. PLoS One 15:e0231965. https://doi.org/10.1371/journal.pone.0231965.

Othman DSMP, Schirra H, McEwan AG, Kappler U (2014) Metabolic versatility in *Haemophilus influenzae*: a metabolomic and genomic analysis. Front Microbiol 5:69. https://doi.org/10.3389/fmicb.2014.00069

Ray A, Lindahl E, Wallner B (2012) Improved model quality assessment using ProQ2. BMC Bioinformatics 13:224. https://doi.org/10.1186/1471-2105-13-224

Schleicher L, Trautmann A, Stegmann DP, Fritz G, Gätgens J, Bott M, Hein S, Simon J, Seifert J, Steuber J (2021a) A sodium-translocating module linking succinate production to formation of membrane potential in *Prevotella bryantii*. Appl Environ Microbiol 87:e0121121. https://doi.org/10.1128/AEM.01211-21

Schleicher L, Herdan S, Fritz G, Trautmann A, Seifert J, Steuber J (2021b) Central carbon metabolism, sodium-motive electron transfer, and ammonium formation by the vaginal pathogen *Prevotella bivia*. Int J Mol Sci 22. https://doi.org/10.3390/ijms222111925

Söding J (2005) Protein homology detection by HMM-HMM comparison. Bioinformatics 21:951–960. https://doi.org/10.1093/bioinformatics/bti125

Takahashi N, Kalfas S, Yamada T (1994) The role of the succinate pathway in sorbitol fermentation by oral *Actinomyces viscosus* and *Actinomyces naeslundii*. Oral Microbiol Immunol 9:218–223. https://doi.org/10.1111/j.1399-302x.1994.tb00061.x.

Vetriani C, Voordeckers JW, Crespo-Medina M, O'Brien CE, Giovannelli D, Lutz RA (2014) Deep-sea hydrothermal vent Epsilonproteobacteria encode a conserved and widespread nitrate reduction pathway (Nap). ISME 8:1510–1521. https://doi.org/10.1038/ismej.2013.246

Weingarten RA, Taveirne ME, Olson JW (2009) The dual-functioning fumarate reductase is the sole succinate:quinone reductase in *Campylobacter jejuni* and is required for full host colonization. J Bacteriol 191:5293–5300. https://doi.org/10.1128/JB.00166-09.
